# A Digital Peer Support Platform to Translate Online Peer Support for Emerging Adult Mental Well-being: Randomized Controlled Trial

**DOI:** 10.2196/43956

**Published:** 2023-04-18

**Authors:** GeckHong Yeo, Gladys Loo, Matt Oon, Rachel Pang, Dean Ho

**Affiliations:** 1 N.1 Institute for Health National University of Singapore Singapore Singapore; 2 The Institute for Digital Medicine (WisDM) Yong Loo Lin School of Medicine National University of Singapore Singapore Singapore; 3 National University of Singapore Singapore Singapore; 4 Acceset Singapore Singapore; 5 Department of Biomedical Engineering College of Design and Engineering National University of Singapore Singapore Singapore; 6 Department of Pharmacology Yong Loo Lin School of Medicine National University of Singapore Singapore Singapore

**Keywords:** mental health, digital health, peer support intervention, peer emotional disclosure, randomized controlled trial

## Abstract

**Background:**

Emerging adulthood (ages 19 to 25 years) is a developmental phase that is marked by increased mental health conditions, especially depression and anxiety. A growing body of work indicates that digital peer emotional support has positive implications for the psychological functioning of emerging adults. There is burgeoning interest among health care professionals, educational stakeholders, and policy makers in understanding the implementation and clinical effectiveness, as well as the associated mechanism of change, of digital peer support as an intervention.

**Objective:**

This randomized controlled trial (RCT) examined the effectiveness of a digital peer support intervention over a digital platform—Acceset—for emerging adult psychological well-being with 3 primary aims. First, we evaluated the implementation effectiveness of digital peer support training for individuals providing support (befrienders) and of the digital platform for peer support. Second, we assessed the clinical outcomes of digital peer support in terms of the intervening effect on emerging adult psychological well-being. Third, we investigated the mechanism of change linking the digital peer support intervention to emerging adult psychological well-being.

**Methods:**

This RCT involving 100 emerging adults from the National University of Singapore follows the published protocol for this trial.

**Results:**

This RCT found effectiveness in digital peer support training—specifically, befrienders’ peer support responses demonstrating significantly higher post- than pretraining scores in selfhood (posttraining score: mean 62.83, SD 10.18, and SE 1.72; pretraining score: mean 54.86, SD 7.32, and SE 1.24; *t*_34_=3.88; *P*<.001). The digital peer support intervention demonstrated clinical effectiveness in enhancing selfhood, compassion, and mindfulness and lowering depressive and anxiety symptoms among seekers in the intervention group after the intervention (mean 7.15, SD 5.14; SE 0.88) than among seekers in the waitlist control group before the intervention (mean 11.75, SD 6.72; SE 0.89; *t*_89_=3.44; *P*<.001). The effect of the intervention on seekers’ psychological well-being was sustained beyond the period of the intervention. The mechanism of change revealed that seekers’ engagement with the intervention had both immediate and prospective implications for their psychological well-being.

**Conclusions:**

This RCT of a digital peer support intervention for emerging adult psychological well-being harnesses the interventional potential of 4 components of psychological well-being and elucidated a mechanism of change. By incorporating and validating the digital features and process of a peer support platform, our RCT provides the parameters and conditions for deploying an effective and novel digital peer support intervention for emerging adult psychological well-being in real-world settings.

**Trial Registration:**

ClinicalTrials.gov NCT05083676; https://clinicaltrials.gov/ct2/show/NCT05083676

## Introduction

### Background and Rationale

Emerging adulthood is a development phase that spans ages 19 to 25 years in most high-income economies [1]. This developmental period is characterized by heightened fluctuations in positivity (ie, the degree to which individuals experience positive emotions) and negativity (ie, the degree to which individuals experience negative emotions) [1]. These normative emotional fluctuations are associated with greater propensity for anxiety and depression, which establishes mental health as a pressing concern among emerging adults [1]. The onset of the global pandemic has resulted in increased distress and systematic changes in positivity and negativity among college students [2,3]. They experienced a decrease in positivity and an increase in negativity that were associated with greater depressive symptoms and anxiety [2,3].

COVID-19’s social implications, which involve uncertainty, insecurity, and a reduced sense of agency and self-directedness, relate most directly to emerging adulthood [4]. The prolonged isolation and reduced social connections brought about by the pandemic have driven college students’ social connections on the web [5,6]. In particular, their use of digital platforms for peer emotional sharing helped them manage intensifying concerns and intense negative feelings associated with the pandemic [7]. There is accumulating evidence underscoring the importance of peers in providing remote emotional support for emerging adults that bears on their psychological functioning [8-10]. Consequently, there is burgeoning interest in understanding digital peer emotional disclosure and support as an intervention, evaluating relevant clinical outcomes, and assessing the associated mechanisms of change [11]. To this end, this randomized controlled trial (RCT) assessed the efficacy of digital peer emotional disclosure and support systems as well as the mechanism of change.

### Digital Peer Support Intervention for Psychological Well-being

#### Overview: The Role of Emotional Disclosure

According to the social sharing of emotions framework, emotional disclosure refers to the interpersonal communication of important and personal experiences with mild to strong positivity and negativity with one’s close social network (eg, “I was exhilarated to receive a good grade on an exam” or “I was distressed being involved in a car accident”) [12,13]. It is a psychological mechanism that facilitates emotional regulation and recovery [12,13]. Theoretically, emotional sharing has important psychoemotional and social implications [12,13]. Emotional sharing maintains one’s exposure to the emotional content and establishes emotional closeness and bonds among sharing partners [12,13]. Specifically, sharing negative emotions helps individuals deal with mental rumination and intrusive thoughts and search for meanings to integrate these experiences into one’s existing narrative. Negative emotions also stimulate social interactions to garner emotional support [12,13]. In contrast, positive emotional experiences facilitate goal achievement by providing positive feedback on successful experiences that can be integrated into one’s knowledge repertoire. Positive emotional episodes represent opportunities for capitalization of positive emotions that enhance one’s positive affect and social bonds [12,13].

The social sharing of emotions framework was originally developed to understand emotional sharing in the offline context, particularly face-to-face interactions [12], and Rime [13] has also extended the framework to the web-based context. Applying the social sharing of emotions framework, scholars have found that college students share emotions on web-based platforms and such sharing may have similar psychological implications as that documented in face-to-face interactions [14,15]. Owing to the affordances of digital platforms, such as reduced nonverbal cues and high accessibility (ie, content is easily available and shared across physical locations [16]), individuals are less apprehensive and able to share emotions with a large audience unconfined by locality. Furthermore, users can elicit immediate feedback and amplify experiences on digital platforms [16]. These features can create a climate of emotional bonding and help individuals manage emotional load, enhance social connections and support, and contribute to social integration [12,17]. An extensive body of work with college students has demonstrated that peers are the primary targets for emotional disclosure [18]. Thus, digital peer emotion sharing has the potential to increase emotion sharers’ sense of social support and the reassurance they are seeking [12,17], especially among college students, at scale.

#### Effective Peer Support

For digital peer support to be effective in mitigating emerging adults’ psychological distress, including anxiety and depression, existing literature underscores the role of 4 components [19-21]. They are (1) enhancing one’s sense of mattering (the extent to which we are important to the surrounding world and people), (2) strengthening selfhood (one’s sense of identity), (3) exploring compassion (the degree of sensitivity to one’s and others’ pain and distress, with a desire to alleviate that distress), and (4) cultivating mindfulness (paying attention to the present moment with intention and acceptance; refer to the *Methods* section for details). Research has revealed that cultivating mattering and selfhood through meaningful and intimate relationships involving peer support has positive implications for young people’s mental well-being [22,23]. For instance, Marshall and Tilton-Weaver [23] reported that mattering to one’s close social networks, specifically peers, that is cultivated through interacting with and caring for them reduces symptoms of depression and anxiety.

As a result of the social implications of the global pandemic, there has been a corresponding surge in research to understand the role of compassion and mindfulness in relation to individuals’ psychological well-being [20,24]. Recent compassion- and mindfulness-based web-based interventions to support college students’ mental health in coping with COVID-19 conditions have demonstrated positive outcomes, including clinical efficacy [20]. Students reported significantly lower levels of stress and anxiety coupled with enhanced levels of self-compassion. These findings provide evidence of the interventional potential of these 4 components in digital peer support. Thus, building college students’ capacity to deliver these components to provide effective support may enhance their peers’ psychological well-being.

Our review of existing literature revealed a limited understanding of how peer emotional disclosure on digital platforms functions as an intervention in support of emerging adults’ psychological well-being [25-27]. The administration of peer support in the offline context, specifically over face-to-face interactions, intervening in various psychological and health outcomes among college students is well documented [28]. Traditional (offline) peer support interventions have demonstrated consistent evidence for implementation and feasibility outcomes, user acceptability, and clinical effectiveness in mediating the psychological well-being of emerging adults, particularly college students [28]. However, with regard to assessing peer support interventions for the mental health of young people, it is important to consider the mechanism of change to elucidate whether and how the intervention affects the development of psychological symptoms—how they unfold over time among emerging adults.

With the onset of the pandemic, the increase in web-based social connections among college students has underscored digital peer emotional disclosure as an intervention that may address specific areas of well-being involving anxiety, depression, and suicidal ideation [28]. As a result, there is burgeoning interest and impetus among researchers, education stakeholders, and health care professionals in establishing the role of peer support on digital platforms and evaluating its clinical effectiveness in improving the psychological well-being of emerging adults, particularly college students [26,27]. Establishing a change model provides the basis for building the therapeutic potential of digital peer support that harnesses the 4 components of emerging adult mental well-being to reduce psychological symptoms. Such findings may provide actionable knowledge for timely and relevant digital peer support for emerging adults’ psychological well-being.

### This Study

#### Overview

Following the published protocol [29], this RCT examined the effectiveness of a digital peer support intervention for the psychological well-being of emerging adults by establishing evidence for clinical outcomes (refer to the study by Yeo et al [29] for details of the trial). In brief, this RCT validated the applicability of harnessing the mattering, selfhood, compassion, and mindfulness components in a digital peer support intervention to promote emerging adults’ mental well-being. By evaluating the mechanism of change involving digital peer support and emerging adults’ psychological functioning, this RCT provided insights into whether or how digital peer support intervened in the development of psychological symptoms. These findings may provide actionable knowledge for timely digital peer support for intervening effectively in emerging adults’ psychological functioning.

#### Aims, Research Questions, and Hypotheses

This study had 2 primary aims. First, we evaluated the digital peer support intervention in terms of training effectiveness for emerging adults providing support (befrienders), who received training in digital peer support skills from a clinical psychologist and certified counselors (moderators). Second, we investigated the clinical effectiveness for the emerging adults who anonymously share emotional experiences to seek support (seekers)—whether it enhanced the 4 components of psychological well-being, specifically, mattering, selfhood, compassion, and mindfulness, and improved the psychological well-being of emerging adults—and the associated mechanism of change linking the digital peer support intervention to emerging adult psychological well-being. As a secondary aim, we also examined the implementation outcomes in terms of feasibility and acceptability of the intervention in offering an ongoing mechanism of support and in identifying individuals with a high risk of having a mental health condition (refer to Multimedia Appendix 1).

To address the first aim, we examined the research question and hypothesis outlined in Textbox 1.

For the second aim, we investigated the research questions and hypotheses outlined in Textbox 2.

**Research question and hypothesis for the first aim of this study.**

- Research question 1
  Is the digital peer support training effective in building befrienders’ capacity to provide effective peer support that harnesses the interventional potential of the 4 components of psychological well-being (mattering, selfhood, compassion, and mindfulness) peer support?
- Hypothesis 1
  The digital peer support training is effective, as shown by the befrienders’ adoption of and fidelity to the training curriculum that applies the 4 components of psychological well-being in providing effective peer support.

**Research questions and hypotheses for the second aim of this study.**

- Research question 2a
  Does the digital peer support intervention enhance the 4 components of psychological well-being among seekers?
- Hypothesis 2a
  The digital peer support intervention increases the 4 components of psychological well-being among seekers over the course of the study across 4 time points.
- Research question 2b
  Is the digital peer support intervention effective in improving emerging adult psychological well-being?
- Hypothesis 2b 
  The digital peer support intervention leads to significantly greater psychological well-being of seekers in the intervention group, as indexed by their lower symptoms of anxiety and depression compared with the waitlist control group. This effect is sustained beyond the period of the intervention at follow-up assessments on all seekers (carryover effects).
- Research question 2c
  What is the mechanism explaining the change in seekers’ psychological well-being in relation to digital peer support?
- Hypothesis 2c
  The initial level and rate of change (ie, growth factors) of befrienders’ online support predict positively the growth factors of seekers’ psychological well-being.

## Methods

### Trial Design

This trial is registered with the US National Library of Medicine ClinicalTrials.gov (NCT05083676). This prospective interventional study evaluated both the implementation of the digital peer support intervention (eg, fidelity, adoption, and utility) and the clinical outcomes (eg, anxiety and depressive symptoms) via an RCT. The seekers (n=50), befrienders (n=30), and moderators (n=2) in arm 1 engaged in the digital peer support intervention (Acceset platform) for 3 weeks, and the control group (n=50) in arm 2 was wait-listed for the intervention. The 4–time-point questionnaire battery on psychological well-being for arm 1 was compared with that for arm 2.

### Ethics Approval

The study protocol received National University of Singapore (NUS) Institutional Ethics Review Board (IRB) approval in October 2021 (NUS-IRB reference S-20-144).

### Intervention

**Figure 1:**
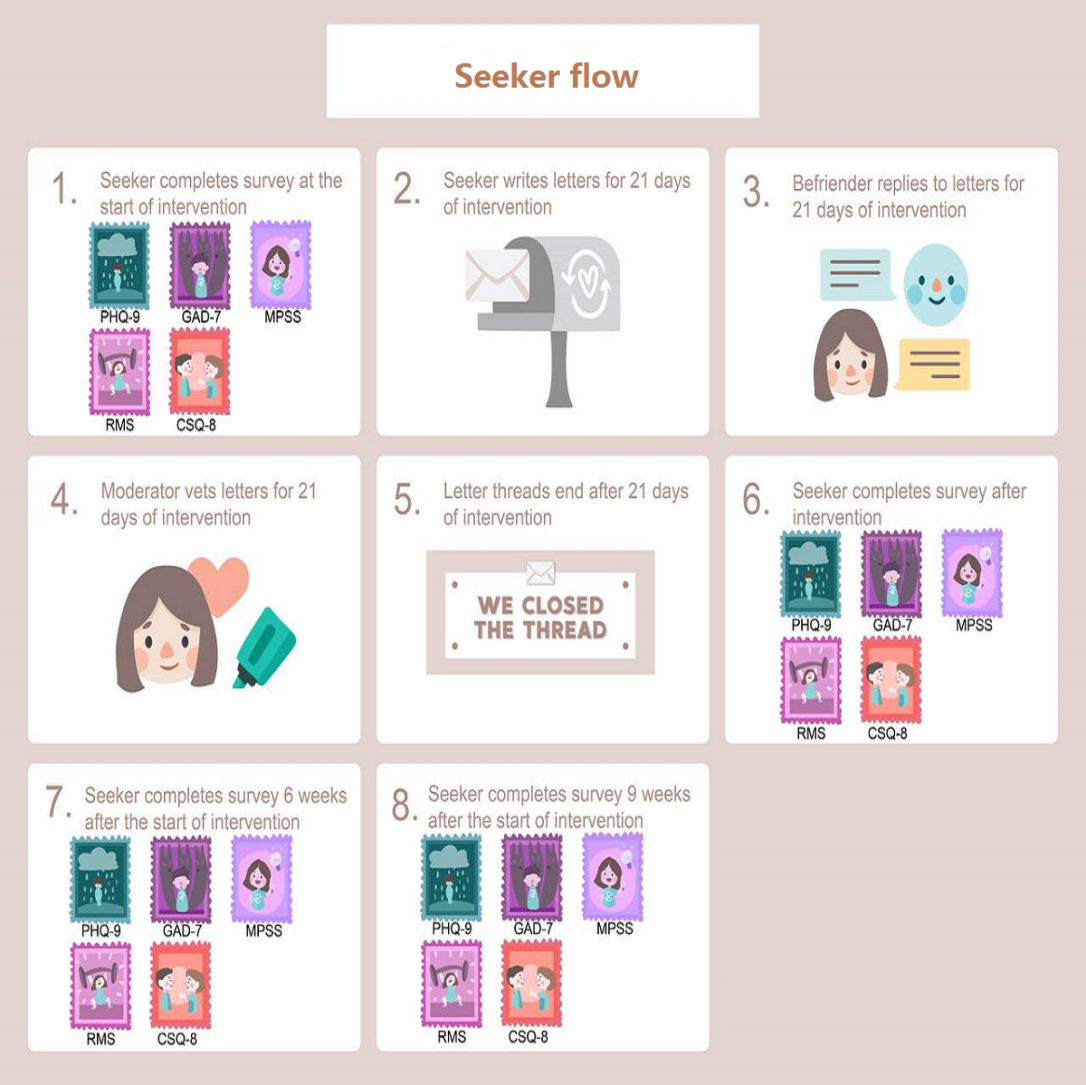
Diagrammatic flow or procedure of the intervention. CSQ-8: 8-item Client Satisfaction Questionnaire; GAD-7: 7-item Generalized Anxiety Disorder Scale; MPSS: Multidimensional Scale of Perceived Social Support; PHQ-9: 9-item Patient Health Questionnaire; RMS: Rapid Mood Screener.

In this study, we engaged Acceset—a stand-alone digital peer support platform that uses a digital text-based intervention involving peer disclosure for emerging adult mental well-being. Acceset incorporates characteristics of successful interventions that affect the psychological well-being of emerging adults [30,31]. Through Acceset, users (seekers) can anonymously share their emotional experiences and receive support from their peers (befrienders). These peers receive training in digital peer support skills from a clinical psychologist and certified counselors (moderators). Acceset digital peer support training for this trial intended to harness mattering, selfhood, compassion, and mindfulness—4 components that reduce emerging adults’ anxiety and depression [19-21]. The Acceset platform incorporates a collection of digital features such as emotion stamps, motivation Graphics Interchange Format emotionality, and functional adjustment stickers as markers of psychological well-being, including emotionality (ie, positivity and negativity), motivations, and functional adjustment (ie, internalizing and externalizing behaviors), respectively. During the course of the intervention, user engagement with these features on the platform served as a source of self-reported information on their psychological well-being status. The Acceset text-based peer disclosure process begins when seekers engage with the platform to seek support with managing their emotional experiences. Details on the Acceset digital peer support training, the platform, and the seeker-befriender interaction (peer support workflow; Figure 1) are provided in the protocol that outlined the RCT [29].

### Sample

#### Befrienders

Participants comprised emerging adults from NUS [29] (mean age 19.60, SD 0.63 years; 17/30, 57% female). The ethnic composition of the sample (23/30, 77% Chinese; 4/30, 13% Malay; 2/30, 7% Indian; and 1/30, 3% others) approximated the Singapore population (74%, 13%, and 12%, respectively [32]). On the basis of participant-reported monthly family income, most participants were from low-middle–income families (<SGD 3000 [US $2224.28]; 13/30, 43%), and the rest were from middle-high–income (>SGD 4000 [US $2965.71]; 10/30, 33%) and middle-income (SGD 3000 to SGD 4000 [US $2224.28-$2965.71]; 7/30, 23%) families. Almost two-thirds of the participants (20/30, 67%) were first-generation college students, and approximately half of them (16/30, 53%) resided at home with parents, with the rest living in campus housing (13/30, 43%) or having an alternative housing arrangement such as an off-campus rental apartment (1/30, 3%).

#### Seekers in the Treatment Arm

Participants (n=291; mean age 19.44, SD 1.28 years; 16/30, 53% female; 24/30, 80% Chinese; 3/30, 10% Malay; 2/30, 7% Indian; and 1/30, 3% others) were emerging adults from NUS [29]. The ethnic composition of the sample was similar to the Singapore population [32]. On the basis of participant-reported monthly family income, most of them were from middle-high–income families (>SGD 4000 [US $2965.71]; 16/30, 53%), and the rest were from middle-income (SGD 3000 to SGD 4000 [US $2224.28-$2965.71]; 8/30, 27%) and low-middle–income (<SGD 3000 [US $2224.28]; 6/30, 20%) families. Slightly more than half (17/30, 57%) of the participants were first-generation college students, and a similar number resided at home with parents (17/30, 57%), with the rest living in campus housing (11/30, 37%) or having an alternative housing arrangement such as an off-campus rental apartment (2/30, 7%).

#### Seekers in the Control Arm

Participants (mean age 19.43, SD 0.66 years; 14/30, 47% female) were emerging adults from NUS [29]. A total of 80% (24/30) of them were Chinese, 7% (2/30) were Malay, 7% (2/30) were Indian, and 7% (2/30) were of other races. These figures were comparable with the ethnic composition of the Singapore population [32]. In total, 63% (19/30) of the participants were first-generation college students. Most of them (26/30, 87%) were living in campus housing, with the rest residing at home with parents (2/30, 7%) or having an alternative housing arrangement such as an off-campus rental apartment (2/30, 7%). On the basis of participant-reported monthly family income, most of them were from middle-income families (>SGD 4000 [US $2965.71]; 14/30, 47%), and the rest were from low-middle– (SGD 3000 to SGD 4000 [US $2224.28-$2965.71]; 7/30, 23%) and middle-high–income (>SGD 4000 [US $2965.71]; 9/30, 30%) families.

The following details are listed in the published protocol that outlined the trial [29]: (1) study setting, (2) eligibility criteria, and (3) peer support workflow.

### Measures

#### The 4 Components of Psychological Well-being (Mattering, Selfhood, Compassion, and Mindfulness)

For each letter exchange between befrienders and seekers, 2 undergraduate assistants extracted and coded the letter content on 4 components. A total of 192 letter exchanges over the 3-week intervention were extracted and coded. On average, 4.67 letters (SD 1.43) were exchanged per day. These letter exchanges were deidentified by the first author and assigned to a team of 2 undergraduate assistants who were trained on the coding procedure outlined in the study by Krippendorff [33] over 1 month until they consistently exceeded adequate reliability standards (κ>0.6 on all variables of interest; refer to Table S1 in Multimedia Appendix 1 for the coding process and samples of participants’ extracted letter exchanges). All codes presented in the following paragraphs were coded independently. The κ coefficients ranged from 0.70 to 0.85, which indicated good interrater reliability.

To determine mattering, coders used the Rosenberg Mattering Scale with 5 items ([34]; eg, “how important are you to others?” and “How interested are others in what you have to say?”) to assess the degree to which befrienders’ responses indicated how important seekers are to others and the degree to which seekers' responses indicated how important they are to others. To assess selfhood, coders rated the extent to which befrienders’ responses built seekers’ self-knowledge, interpersonal self, and self-agency using the Rosenberg Global Self-Esteem Scale ([35]; eg, “the peer is a person of worth, at least on an equal plane with others” and “the peer has a number of good qualities”), the Self-Consciousness Scale ([36]; eg, “the peer is constantly examining his/her motives” and “the peer is concerned about what other people think of him/her”), the General Self-Efficacy Scale ([37]; eg, “When the peer makes plans, he/she is certain that he/she can make them work” and “the peer is a self-reliant person”), and the Social Self-Efficacy Scale ([38]; eg, “The peer does not handle himself/herself well in social gatherings” and “It is difficult for the peer to make new friends”). For seekers, coders rated the extent to which their emotional disclosure reflected self-knowledge, interpersonal self, and self-agency using the same subscales and items except with a different item stem (eg, “he/she is a person of worth, at least on an equal plane with others” and “he/she has a number of good qualities”). A composite selfhood score was obtained by averaging the ratings across all 4 selfhood scales for both befrienders and seekers.

The compassion of befrienders’ peer support responses and seekers’ peer emotional disclosure was assessed by coders using the Compassion Cultivation Training program [37] as reflecting (1) an awareness of distress, (2) affective concern, and (3) a responsiveness or readiness to help relieve that distress (motivational). Mindfulness was measured as a flexible cognitive state in which individuals are actively present and notice novel aspects in both the environment and one’s perspectives [39]. Befrienders’ support responses and seekers’ peer emotional disclosure were evaluated based on the degree to which they harnessed mindful self-acceptance: (1) identifying novel aspects of the situation or perspective; (2) demonstrating “work in progress” by using possibility words such as “could be” and offering other interpretations of the situation; (3) highlighting puzzles and paradoxes, for example, how individuals may feel victimized yet they are responsible for being in that situation; (4) noticing humorous aspects of the situation; (5) perceiving the situation from multiple perspectives; (6) considering alternative (useful) aspects of a problematic context or the silver lining; (7) emphasizing a mental file of positive memories; and (8) encouraging mindfulness journaling.

To assess mattering, selfhood, compassion, and mindfulness, a 4-point scale ranging from 1 (not at all) to 4 (a lot) was used by raters. On the basis of raters’ scores, the Cronbach α for the befrienders’ mattering was .86 for the first letter exchange, .84 for the second letter exchange, .87 for the third letter exchange, and .91 for the fourth letter exchange. For the seekers’ mattering, the Cronbach α was .83, .82, .77, and .74 for the first, second, third, and fourth letter exchanges, respectively. The Cronbach α for the befrienders’ selfhood was .77 for the first letter exchange, .76 for the second letter exchange, .71 for the third letter exchange, and .70 for the fourth letter exchange. For the seekers’ selfhood, the Cronbach α was .85, .88, .89, and .90 for the first, second, third, and fourth letter exchanges, respectively.

The Cronbach α for the befrienders’ compassion was .88 for the first letter exchange, .71 for the second letter exchange, .74 for the third letter exchange, and .75 for the fourth letter exchange. For the seekers’ compassion, the Cronbach α was .88, .91, .90, and .91 for the first, second, third, and fourth letter exchanges, respectively. The Cronbach α for the befrienders’ mindfulness was .76 for the first letter exchange, .80 for the second letter exchange, .73 for the third letter exchange, and .70 for the fourth letter exchange. For the seekers’ mindfulness, the Cronbach α was .84, .88, .89, and .88 for the first, second, third, and fourth letter exchanges, respectively. The Cronbach α demonstrated adequate internal consistency in this study, similar to that established in other Asian contexts such as China—mattering (Cronbach α=.69 [40]), selfhood (self-esteem: Cronbach α=.85; self-efficacy: Cronbach α=.82; self-consciousness: Cronbach α=.6 [41,42]), compassion (Cronbach α=.88 [43]), and mindfulness (Cronbach α=.85 [43]).

#### Befrienders’ Peer Support Responses

We tested 1-factor versus 4-factor models for befrienders’ support (with the 4 components of psychological well-being as indicators measuring a latent construct). Results indicated acceptable to excellent fit of the 1-factor model, which was a better fit than the 4-factor model, across the 4 time points (refer to Table S2 in Multimedia Appendix 1). Thus, we created a composite peer support score with mattering, selfhood, compassion, and mindfulness scales as a set of metrics for assessing befrienders’ peer support responses for each of the 4 letter exchanges, with higher scores representing greater peer support.

#### Psychological Well-being (Times 1-4)

We created a latent construct with 2 indicators—anxiety and depression. Befrienders and seekers provided self-report responses to the 7-item General Anxiety Disorder Scale (GAD-7), which provides rapid screening for the presence of clinically significant anxiety symptoms, particularly in outpatient settings ([44]; eg, “feeling nervous, anxious, or on edge” and “not being able to stop or control worrying”), and the 9-item Patient Health Questionnaire (PHQ-9), which is a 9-item scale that quantifies depressive symptoms and monitors their severity ([45]; eg, “little interest or pleasure in doing things” and “feeling down, depressed or hopeless”). All the items on the GAD-7 and PHQ-9 were rated on a 4-point scale ranging from 0 (not sure at all) to 4 (nearly every day) across 4 time points: time 1 (before the intervention), time 2 (postintervention assessment; 3 weeks), time 3 (first follow-up; 6 weeks), and time 4 (second and final follow-up; 9 weeks).

We tested a 1-factor versus a 2-factor model for the seekers’ psychological well-being (with the GAD-7 and PHQ-9 items as indicators measuring a latent construct). Results indicated acceptable to excellent fit of the 1-factor model, which was a better fit than the 2-factor model, across the 4 time points (refer to Table S2 in Multimedia Appendix 1). Thus, we created a composite psychological well-being score using the GAD-7 and PHQ-9 scores as a metric for assessing psychological well-being, with lower scores representing greater well-being. The Cronbach α for seekers’ psychological well-being was .88 at time 1 (before the intervention), .94 at time 2 (postintervention assessment; 3 weeks), .93 at time 3 (first follow-up; 6 weeks), and .95 at time 4 (second and final follow-up; 9 weeks). The GAD-7 and PHQ-9 have demonstrated adequate internal consistency in Asian contexts such as China (Cronbach α=.85 and .89, respectively [46]).

#### Perceived Social Support (Times 1-4)

Seekers responded to the 12-item Multidimensional Scale of Perceived Social Support, which assessed perceived social support from 3 sources—family, friends, and significant others ([47]; eg, “There is a special person who is around when I am in need” and “I get the emotional help and support I need from my family”)—using a 7-point scale (1=very strongly disagree; 7=very strongly agree). The Cronbach α for seekers’ perceived social support was .88 at time 1 (before the intervention), .90 at time 2 (postintervention assessment; 3 weeks), .94 at time 3 (first follow-up; 6 weeks), and .93 at time 4 (second and final follow-up; 9 weeks). The Multidimensional Scale of Perceived Social Support has demonstrated adequate internal consistency in Asian contexts such as China (Cronbach α=.86 [48]).

### Data Management and Planned Analyses

#### Overview

Consistent with the procedure for data management and planned analyses outlined in the study by Yeo et al [29], Acceset provided a cloud storage for depositing data on participants’ responses to all the measures as well as written letters between seekers and befrienders on the Acceset platform. Following the NUS-IRB protocol, each participant had a nonidentifying unique ID, and all data were deidentified before analyses, conducted by the lead author and 2 student interns. On the basis of a power analysis conducted before the commencement of the RCT, 50 participants each for the intervention and waitlist control groups were needed to find significant effects with the total number of outcomes examined in the RCT. Using a Monte Carlo stimulation [49,50], our power analysis on the mechanism of change indicated that 100 participants provided 80% power to detect significant linkages between support from befrienders and psychological well-being of seekers based on criteria listed in the studies by Muthén and Muthén [49,50]. Our power revealed that the range of biases of the parameter and SE (0.04% to 1.40%), SE (0.05% to 1.72%), and coverage (0.94 to 1.00) met the criteria for power to approximate or exceed 80% listed in the studies by Muthén and Muthén [49,50]. For instance, the parameter and SE biases do not exceed 10% for all parameters in the model. We performed all analyses using RStudio (Posit) [51].

#### Aim 1

To address research question 1 and hypothesis 1, a total of 2 student assistants conducted qualitative analyses of all letter exchanges between befrienders and seekers by extracting, coding, and evaluating the degree to which befrienders’ support responses reflected the 4 components of psychological well-being—mattering, selfhood, compassion, and mindfulness. We then examined if befrienders’ pre- and posttraining peer support responses were significantly different using the independent-sample *t* test (2-tailed) with Bonferroni post hoc tests to control for multiple comparisons.

#### Aim 2

For research question 2a and hypothesis 2a, we ascertained whether the intervention increased seekers’ experience of the 4 components of psychological well-being by performing 4 sets of latent growth curve modeling to examine the changes in mattering, selfhood, compassion, and mindfulness. To model baseline mean levels and rates of change (measured as intercepts and slope factors, respectively), we used repeated-measure data comprising the 4 time points of a given composite score as indicators of latent variables. In each of the 4 baseline latent growth models (ie, mattering, selfhood, compassion, and mindfulness), we estimated and compared the linear, quadratic, and nonlinear curve-fitting models. In all 4 models, the loadings of the intercept factor were constrained to 1 on each time point. In the linear model, the factor loadings for slope were set at 0, 1, 2, and 3; in the quadratic model, the factor loadings were set at 0, 1, 4, and 9; and in the nonlinear curve-fitting model, the factor loadings were set at 0 and 1 for time 1 and time 2, respectively, and freely estimated for times 3 and 4.

In evaluating each model, we examined the following fit indexes: the chi-square statistic, the root mean square error of approximation (RMSEA), the standardized root mean square residual (SRMR), and the comparative fit index (CFI). Acceptable model fit was indicated by an RMSEA of <0.08 (90% CI 0.05-0.10), an SRMR with values of ≤1.0, and a CFI of >0.90. RMSEA and SRMR values of <0.05 (90% CI 0.05-0.10) and CFI values of >0.95 were considered a good fit [52]. The linear model was nested within the quadratic and optimal-fit models, and the quadratic and optimal-fit models were not nested. Thus, the Bayesian information criterion (BIC) was used for model comparisons [53].

As for research question 2b and hypothesis 2b, we investigated whether the intervention affected seekers’ psychological well-being using independent-sample *t* tests (2-tailed) with Bonferroni post hoc tests. First, seekers’ experience of anxiety and depressive symptoms in the intervention group at postintervention assessment (week 3) was compared with the symptoms of the waitlist control group before the intervention (week 0). Second, we assessed the sustained effects of the intervention on the psychological well-being of all seekers (intervention and waitlist) by comparing baseline symptoms (week 0) with postintervention symptoms (week 3), as well as symptoms at each follow-up (weeks 6 and 9). Similarly, we assessed seekers’ support-seeking behaviors by comparing their perceived social support at baseline (week 0) with that at the postintervention (week 3) and follow-up (weeks 6 and 9) assessments.

We addressed research question 2c and hypothesis 2c on the mechanism of change by evaluating a conditional growth model on befrienders’ peer support predicting seekers’ psychological well-being. Befrienders’ sex was included as a covariate as female individuals are more likely to provide support than male individuals [54]. The seekers’ sex and socioeconomic status (ie, monthly household income) were included as covariates as female students and those of low socioeconomic status are more likely to experience anxiety and depression [54]. Following the procedure outlined in the study by Kaplan [53], a random sample of 20 participants was used to plot and explore separately the variability in the level and trend of befrienders’ support and seekers’ psychological well-being in college. We then examined the trajectories of befrienders’ support and seekers’ well-being by fitting individual baseline growth models following the same procedure outlined previously on assessing the trajectories of the 4 components of psychological well-being among seekers. These steps were necessary to prevent problems with specification (ie, negative residual variances) when modeling growths in digital peer support and psychological well-being simultaneously.

## Results

### Preliminary Analyses

The nonsignificant result from the Little Missing Completely at Random test showed that the missing data involving all the variables of interest in this study (refer to the *Measures* section), which ranged from 19% to 58%, were nonsystematic (*χ*^2^_3783_=766.0; *P*>.99). Thus, we handled missing data using full information maximum likelihood imputation [53]. Maximum likelihood imputation is a method that ascertains the parameter values of a model using mean and variance; it maximizes the chance that the values generated are closest to those observed.

We used multiple group confirmatory factor analyses to establish sequentially the different levels of measurement invariance of the 1-factor befriender support and 1-factor seeker psychological well-being models across 4 time points. Results revealed configural invariance (in factorial structure), metric invariance (in factor loadings), and scalar invariance (in item intercepts) with acceptable model fit indexes (RMSEA<0.08; SRMR<0.10; CFI>0.90). Comparing constrained versus unconstrained measurement models, the differences in goodness-of-fit indexes were below the recommended cutoff value of CFI=0.01 [55]. These results confirmed the longitudinal invariance of the 1-factor model for both befrienders’ support and seekers’ psychological well-being for meaningful comparisons of scores across time.

Tables 1 and 2 present the descriptive statistics and correlations among variables for each letter exchange for seekers and befrienders, respectively. Of the 4 components of psychological well-being, mattering and selfhood displayed consistently moderate and positive associations, and similar associations were documented between compassion and mindfulness for both befrienders and seekers. For seekers, perceived social support had low to moderate negative associations with psychological symptoms at each time point (eg, support and well-being at time 1 [before the intervention]) and across time (support at time 1 and well-being at time 3 [first follow-up at 6 weeks]; refer to Table S3 in Multimedia Appendix 1).

**Table 1:** Correlations and descriptive statistics for seekers on study variables for letter exchanges over the 3-week intervention.^a^

	1	2	3	4	5	6	7	8	9	10	11	12	13	14	15	16	17	18	19	Mean (SD)
1. Ma^b^ 1^c^	—^d^	—	—	—	—	—	—	—	—	—	—	—	—	—	—	—	—	—	—	3.12 (1.09)
2. Ma 2^e^	0.26	—	—	—	—	—	—	—	—	—	—	—	—	—	—	—	—	—	—	2.86 (0.87)
3. Ma 3^f^	0.43	0.53	—	—	—	—	—	—	—	—	—	—	—	—	—	—	—	—	—	2.58 (1.02)
4. Ma 4^g^	0.60	0.58	0.78	—	—	—	—	—	—	—	—	—	—	—	—	—	—	—	—	2.56 (0.99)
5. S^h^ 1	0.18	0.12	0.11	0.10	—	—	—	—	—	—	—	—	—	—	—	—	—	—	—	2.47 (1.29)
6. S 2	0.21	0.06	0.18	0.22	0.17	—	—	—	—	—	—	—	—	—	—	—	—	—	—	3.07 (1.76)
7. S 3	0.27	0.13	0.10	0.22	0.14	0.38	—	—	—	—	—	—	—	—	—	—	—	—	—	3.39 (1.05)
8. S 4	0.27	0.27	0.28	0.18	0.21	0.58	0.41	—	—	—	—	—	—	—	—	—	—	—	—	3.63 (1.77)
9. C^i^ 1	0.15	0.26	0.14	0.15	0.28	0.13	0.10	0.24	—	—	—	—	—	—	—	—	—	—	—	2.63 (1.02)
10. C 2	0.10	0.21	0.28	0.32	0.18	0.15	0.11	0.12	0.44	—	—	—	—	—	—	—	—	—	—	3.53 (1.24)
11. C 3	0.15	0.19	0.26	0.33	0.16	0.13	0.22	0.20	0.35	0.70	—	—	—	—	—	—	—	—	—	3.53 (0.75)
12. C 4	0.26	0.29	0.16	0.46	0.29	0.10	0.14	0.24	0.44	0.78	0.70	—	—	—	—	—	—	—	—	3.84 (0.81)
13. M^j^ 1	0.19	0.11	0.16	0.18	0.19	0.16	0.16	0.14	0.16	0.18	0.17	0.22	—	—	—	—	—	—	—	3.30 (1.00)
14. M 2	0.18	0.19	0.11	0.17	0.12	0.13	0.18	0.14	0.18	0.30	0.17	0.13	0.25	—	—	—	—	—	—	3.16 (1.90)
15. M 3	0.11	0.15	0.16	0.17	0.14	0.15	0.19	0.21	0.13	0.15	0.39	0.17	0.45	0.60	—	—	—	—	—	2.91 (1.35)
16. M 4	0.20	0.24	0.17	0.13	0.27	0.21	0.12	0.17	0.26	0.11	0.35	0.30	0.56	0.56	0.64	—	—	—	—	2.31 (1.18)
17. PWB^k^ 1	0.15	0.12	0.34	0.15	0.21	0.11	0.17	0.38	0.12	0.10	0.19	0.16	0.27	0.14	0.20	0.28	—	—	—	10.72 (1.06)
18. PWB 2	0.05	0.38	0.24	0.15	0.24	0.45	0.23	0.20	0.17	0.25	0.26	0.19	0.20	0.21	0.19	0.11	0.52	—	—	9.68 (1.08)
19. PWB 3	0.17	0.33	0.42	0.28	0.32	0.13	0.15	0.14	0.18	0.18	0.11	0.18	0.31	0.17	0.13	0.15	0.54	0.76	—	8.41 (1.12)
20. PWB 4	0.08	0.29	0.36	0.26	0.10	0.33	0.23	0.13	0.13	0.28	0.10	0.13	0.34	0.12	0.25	0.11	0.52	0.69	0.78	8.35 (1.16)

^a^Coefficients above the diagonal refer to those for befrienders, and coefficients below the diagonal refer to those for seekers. All correlation coefficients >0.15 are significant.

^b^Ma: mattering.

^c^1: first letter exchange.

^d^Not applicable.

^e^2: second letter exchange.

^f^3: third letter exchange.

^g^4: fourth letter exchange.

^h^S: selfhood.

^i^C: compassion.

^j^M: mindfulness.

^k^PWB: psychological well-being.

**Table 2:** Correlations and descriptive statistics for befrienders on study variables for letter exchanges over the 3-week intervention.^a^

	1	2	3	4	5	6	7	8	9	10	11	12	13	14	15	16	17	18	19	Mean (SD)
1. Ma^b^ 1^c^	—^d^	—	—	—	—	—	—	—	—	—	—	—	—	—	—	—	—	—	—	3.14 (0.17)
2. Ma 2^e^	0.74	—	—	—	—	—	—	—	—	—	—	—	—	—	—	—	—	—	—	2.91 (0.83)
3. Ma 3^f^	0.46	0.80	—	—	—	—	—	—	—	—	—	—	—	—	—	—	—	—	—	2.59 (0.60)
4. Ma 4^g^	0.89	0.87	0.83	—	—	—	—	—	—	—	—	—	—	—	—	—	—	—	—	2.20 (0.52)
5. S^h^ 1	0.56	0.66	0.35	0.57	—	—	—	—	—	—	—	—	—	—	—	—	—	—	—	2.74 (1.87)
6. S 2	0.30	0.34	0.34	0.34	0.72	—	—	—	—	—	—	—	—	—	—	—	—	—	—	3.43 (1.87)
7. S 3	0.24	0.20	0.50	0.50	0.51	0.26	—	—	—	—	—	—	—	—	—	—	—	—	—	3.68 (1.17)
8. S 4	0.13	0.38	0.14	0.17	0.31	0.35	0.61	—	—	—	—	—	—	—	—	—	—	—	—	3.87 (0.32)
9. C^i^ 1	0.34	0.30	0.27	0.36	0.48	0.16	0.34	0.53	—	—	—	—	—	—	—	—	—	—	—	1.29 (1.97)
10. C 2	0.19	0.66	0.30	0.10	0.37	0.45	0.39	0.29	0.82	—	—	—	—	—	—	—	—	—	—	2.27 (1.20)
11. C 3	0.27	0.10	0.20	0.51	0.21	0.24	0.35	0.35	0.66	0.45	—	—	—	—	—	—	—	—	—	2.49 (0.53)
12. C 4	0.52	0.42	0.16	0.40	0.19	0.20	0.13	0.13	0.40	0.54	0.73	—	—	—	—	—	—	—	—	2.36 (1.13)
13. M^j^ 1	0.35	0.13	0.20	0.30	0.23	0.57	0.11	0.11	0.19	0.27	0.23	0.16	—	—	—	—	—	—	—	1.51 (0.63)
14. M 2	0.24	0.42	0.30	0.49	0.60	0.50	0.15	0.15	0.16	0.47	0.29	0.14	0.85	—	—	—	—	—	—	2.87 (1.59)
15. M 3	0.17	0.50	0.35	0.30	0.49	0.35	0.14	0.14	0.15	0.24	0.21	0.17	0.55	0.63	—	—	—	—	—	3.17 (1.18)
16. M 4	0.18	0.21	0.16	0.37	0.35	0.35	0.17	0.17	0.18	0.28	0.11	0.18	0.58	0.60	0.53	—	—	—	—	3.24 (1.61)
17. PWB^k^ 1	0.12	0.23	0.57	0.15	0.31	0.30	0.48	0.48	0.40	0.68	0.25	0.56	0.27	0.28	0.22	0.41	—	—	—	2.61 (1.33)
18. PWB 2	0.21	0.18	0.54	0.27	0.33	0.34	0.41	0.41	0.55	0.30	0.30	0.39	0.38	0.17	0.38	0.44	0.54	—	—	2.92 (0.74)
19. PWB 3	0.11	0.43	0.57	0.35	0.36	0.15	0.46	0.46	0.76	0.55	0.25	0.56	0.19	0.28	0.23	0.39	0.76	0.79	—	3.25 (1.52)
20. PWB 4	0.10	0.20	0.24	0.10	0.35	0.14	0.48	0.48	0.49	0.34	0.38	0.57	0.13	0.20	0.53	0.49	0.53	0.38	0.66	3.60 (1.62)

^a^All correlation coefficients >0.15 are significant.

^b^Ma: mattering.

^c^1: first letter exchange.

^d^Not applicable.

^e^2: second letter exchange.

^f^3: third letter exchange.

^g^4: fourth letter exchange.

^h^S: selfhood.

^i^C: compassion.

^j^M: mindfulness.

^k^PWB: psychological well-being.

### Main Analyses

#### Aim 1

To address research question 1 and hypothesis 1, we examined if Acceset digital peer support training was effective in training befrienders to provide effective peer support by assessing the adoption of and fidelity to the training curriculum through the letter exchanges between seekers and befrienders. Specifically, we evaluated the text of the letter exchanges in terms of the extent to which befrienders displayed fidelity in applying the 4 components of psychological well-being—specifically, mattering, selfhood, compassion, and mindfulness—in their peer responses to seekers’ disclosures. To control for multiple comparisons, we applied the Bonferroni post hoc tests. Befrienders’ peer responses demonstrated greater selfhood (support) after training (mean 62.83, SE 10.18) than before training (mean 54.86, SE 7.32; *t*_34_=3.88; *P*<.001). However, their support responses did not indicate greater mattering after training (mean 9.03, SE 3.87) than before training (mean 9.34, SE 3.87; *t*_34_=0.40; *P*=.69). The same outcomes were observed for compassion after training (mean 8.57, SE 3.20) and before training (mean 9.09, SE 3.97; *t*_34_=0.40; *P*=.50) and mindfulness after training (mean 10.00, SE 2.63) and before training (mean 11.17, SE 3.59; *t*_34_=1.64; *P*=.11).

#### Aim 2

##### Research Question 2a and Hypothesis 2a

**Figure 2:**
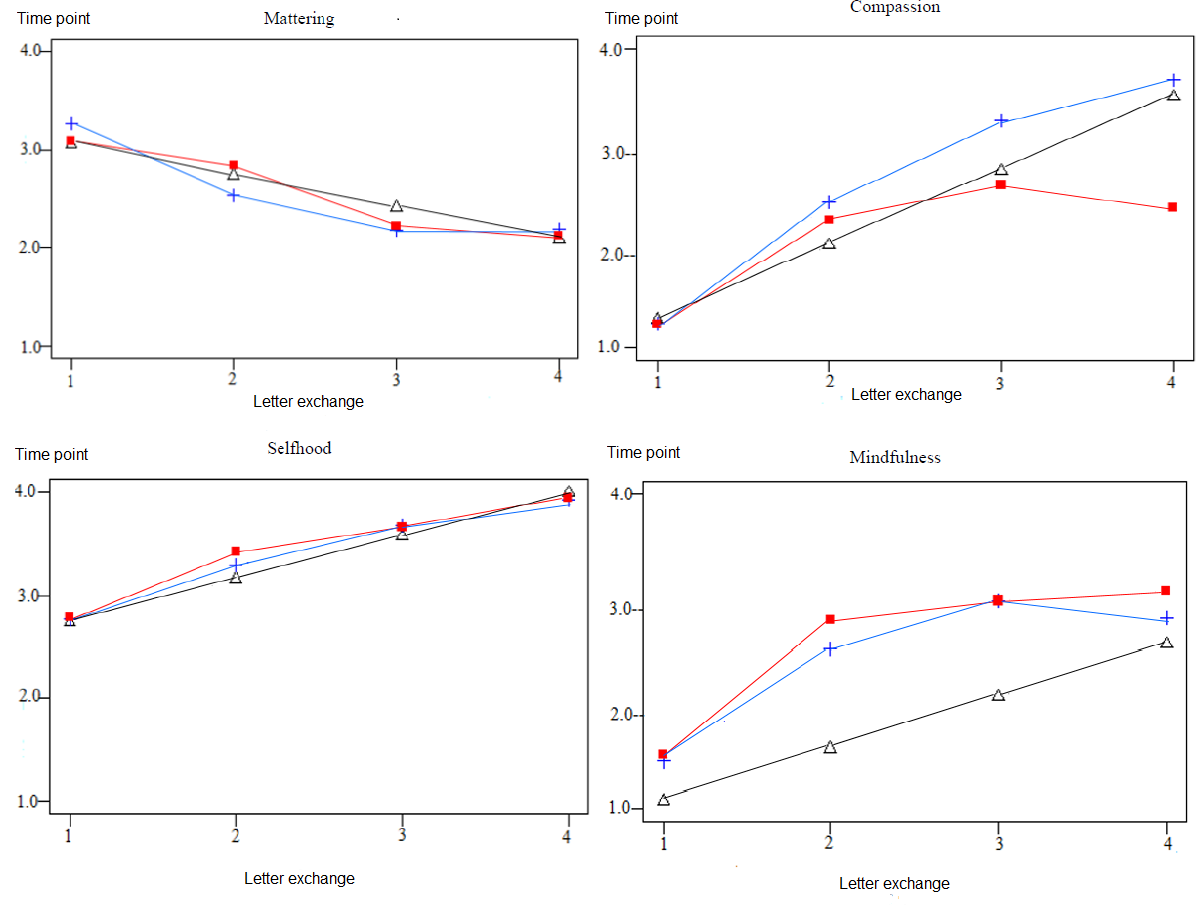
Latent growth curve models for the 4 components of psychological well-being among seekers. For mattering and selfhood, the linear baseline model was the best-fitting model (in black); for compassion and mindfulness, the nonlinear curve-fitting baseline model was the best-fitting model (in red).

To address research question 2a and hypothesis 2a, we examined if the Acceset peer support training of befrienders enhanced the 4 components of psychological well-being among seekers. We compared the change in mattering, selfhood, compassion, and mindfulness scores of seekers over the course of the study. We conducted 4 sets of latent growth curve modeling to examine the trajectories of mattering, selfhood, compassion, and mindfulness by fitting individual baseline growth models. Table 3 presents the parameter estimates and fit statistics for the latent growth curve models for the 4 components of psychological well-being. For each component, we found a best-fit model. The chi-square difference tests, BICs, and the various alternative fit indexes indicated that alternative models were poorer fits. Figure 2 shows the models examined and the best-fit models for each of the 4 components of psychological well-being.

**Table 3:** Latent growth models for the 4 components of psychological well-being: fit indexes and estimates.

Component and model		Goodness of fit						Estimates	
		RMSEA^a^ (90% CI)	CFI^b^	SRMR^c^	BIC^d^	Chi-square (*df*)	Intercept β^e^ (SE)		Slope (SE)
**Mattering**									
	*L* ^f,g^	*0.00 (0.00-0.05)*	*1.00*	*0.04*	*1284.69*	*1.45 (5)*	*3.14* ^h^ *(0.27)*		−*0.48 (0.13)*
	Q^i^	0.02 (0.01-0.05)	0.98	0.08	1288.67	0.91 (4)	2.42^h^ (0.30)		−0.46 (0.42)
	NL^j^	0.04 (0.00-0.09)	0.96	0.09	1292.76	0.39 (3)	3.03^h^ (0.28)		0.13 (0.22)
**Selfhood**									
	*L*	*0.03 (0.00-0.05)*	*0.99*	*0.08*	*836.88*	*5.48 (5)*	*2.74* ^h^ *(0.12)*		*0.42* ^h^ *(0.08)*
	Q	0.04 (0.00-0.16)	0.98	0.09	869.02	4.60 (4)	2.77^h^ (0.12)		0.59^h^ (0.20)
	NL	0.09 (0.00-0.17)	0.90	0.08	871.90	2.87 (3)	2.79^h^ (0.12)		0.60^h^ (0.16)
**Compassion**									
	L	0.22 (0.15-0.30)	0.67	0.44	1748.36	28.74^h^ (5)	1.57^h^ (0.64)		0.49^h^ (0.66)
	Q	0.11 (0.00-0.21)	0.92	0.15	1733.34	9.11^k^ (4)	1.44^h^ (0.61)		0.45^h^ (1.13)
	*NL*	*0.00 (0.00-0.04)*	*1.00*	*0.06*	*1730.62*	*1.79 (3)*	*1.29* ^h^ *(0.01)*		*0.61* ^h^ *(0.91)*
**Mindfulness**									
	L	0.29 (0.22-0.37)	0.00	0.30	1669.95	47.16^h^ (5)	1.49^h^ (0.96)		0.33^h^ (0.62)
	Q	0.17 (0.08-0.26)	0.00	0.20	1642.34	14.95^h^ (4)	1.30^h^ (0.80)		0.23^h^ (0.10)
	*NL*	*0.06 (0.00-0.09)*	*0.92*	*0.06*	*1605.62*	*7.78 (3)*	*1.51* ^h^ *(0.60)*		*0.87* ^h^ *(0.93)*

^a^RMSEA: root mean square error of approximation.

^b^CFI: comparative fit index.

^c^SRMR: standardized root mean square residual.

^d^BIC: Bayesian information criterion.

^e^Standardized estimate.

^f^L: linear.

^g^Italics indicate the best-fit models.

^h^*P*<.01.

^i^Q: quadratic.

^j^NL: nonlinear curve.

^k^*P*<.05.

##### Mattering

The linear model demonstrated excellent fit, but rate of change was not significant. The initial level and rate of change in mattering did not indicate significant association (*r*_100_=0.17; *P*=.12). Seekers’ sense of mattering demonstrated limited change at 0 (baseline), 3 (postintervention assessment), 6 (follow-up assessment), and 9 (follow-up assessment) weeks.

##### Selfhood

The linear model demonstrated excellent fit. The moderate initial level of selfhood increased linearly from baseline to the final follow-up assessment, and the initial level was unrelated to its rate of change (*r*_100_=0.03; *P*=.95). Seekers had moderate level of selfhood development before the intervention, and the increase in selfhood at postintervention assessment was sustained at the follow-up assessments at 6 and 9 weeks.

##### Compassion

The optimal curve-fitting model revealed excellent fit. The low initial level of compassion had a sharp increase from baseline to postintervention assessment, which increased modestly at subsequent follow-up assessments. The initial level of mindfulness was not significantly related to its rate of change (*r*_100_=0.27; *P*=.21). Before the Acceset peer support intervention, seekers indicated a low level of compassion that increased substantially following the intervention and modestly at the 6- and 9-week follow-ups.

##### Mindfulness

The optimal curve-fitting model demonstrated excellent fit and revealed a low initial level with sharp increase following the intervention and continued to increase subsequently but dipped slightly at the last follow-up assessment. The initial level of mindfulness was not significantly related to its rate of change (*r*_100_=0.33; *P*=.56). Seekers experienced a pronounced increase in mindfulness from baseline to postintervention assessment, and this continued to increase at the 6-week follow-up. However, mindfulness decreased slightly at 9 weeks—the final follow-up.

##### Research Question 2b and Hypothesis 2b

As for research question 2b and hypothesis 2b, congruent with our hypothesis, engagement with Acceset digital peer support led to improved mental well-being of seekers in the intervention group, particularly lower psychological symptoms (postintervention assessment; mean 7.15, SE 0.88; Table 4), as compared with the waitlist control group (before the intervention; mean 11.75, SE 0.89; *t*_89_=3.44; *P*<.001). We assessed the sustained effect of the digital peer support intervention beyond the period of the intervention by evaluating the change in mental well-being of the participants in both groups (intervention and control) after 3, 6, and 9 weeks from the baseline using self-report questionnaires. In conducting independent-sample *t* tests, we controlled for multiple comparisons using Bonferroni post hoc tests. Seekers experienced less psychological symptoms at postintervention assessment (ie, 3 weeks; mean 9.49, SE 1.16; *t*_46_=3.14; *P*=.003) and at the first follow-up (ie, 6 weeks; mean 8.45, SE 1.30; *t*_38_=3.20; *P*=.003), but not at the second follow-up (ie, 9 weeks; mean 10.26, SE 1.51; *t*_38_=1.74; *P*=.09), than they did at baseline (mean 12.06, SE 1.20). Seekers’ support-seeking behaviors beyond the Acceset platform were assessed through their perceived social support at weeks 6 and 9. Compared with baseline (mean 63.38, SE 1.50), seekers’ perceived social support did not differ significantly at 3 weeks (mean 63.92, SE 1.35; *t*_62_=0.43; *P*=.67), 6 weeks (mean 62.25, SE 2.09; *t*_54_=0.44; *P*=.66), and 9 weeks (mean 62.60, SE 2.38; *t*_42_=0.46; *P*=.65).

**Table 4:** Descriptive statistics for support and psychological well-being among seekers in the treatment and control arms at baseline, postintervention assessment, and follow-ups.

		Baseline^a^, mean (SD)	Postintervention assessment^b^, mean (SD)	First follow-up^c^, mean (SD)	Second follow-up^d^, mean (SD)
**Treatment**					
	PSS^e^	63.47 (9.71)	66.06 (7.92)	61.35 (9.15)	60.60 (11.20)
	PWB^f^	10.16 (3.22)	7.15 (0.88)	9.71 (2.99)	10.18 (3.45)
**Control**					
	PSS	62.39 (8.99)	61.78 (8.33)	63.15 (9.04)	64.60 (10.31)
	PWB	11.75 (2.97)	8.13 (1.15)	7.19 (1.89)	10.34 (3.99)

^a^First time point (before the intervention; 0 weeks).

^b^Second time point (3 weeks).

^c^Third time point (6 weeks).

^d^Fourth time point (9 weeks).

^e^PSS: perceived social support.

^f^PWB: psychological well-being.

Additional independent *t* tests were conducted to determine if seekers who provided data across all 4 time points were different from those who discontinued the study and provided less data points, which could bias our results. We expected instances when seekers and befrienders dropped out of the study, for example, when seekers indicated high risk of depression and suicidality during the course of the study. It was also possible that befrienders discontinued the letter exchange with seekers because the letter content caused distress to the befrienders. In such cases, the platform informed the seekers and sought their consent to end the letter thread or continue the letter exchange with a new befriender.

**Figure 3:**
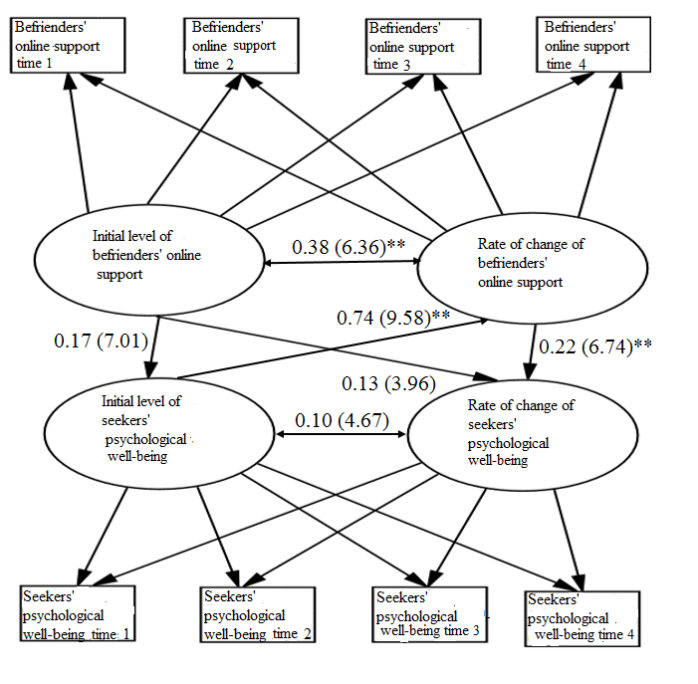
Conditional growth model on befrienders’ support and seekers’ psychological well-being (befrienders: n=30 emerging adults; seekers: n=100 emerging adults). All path coefficients are standardized, with SEs in parentheses; none of the covariates were significant, and they were not included in this model. **P<.01.

For research question 2c and hypothesis 2c, we elucidated the mechanism of change linking the digital peer support intervention to emerging adult mental well-being (Figure 3) by assessing whether and how the initial level and rate of change from baseline to weeks 3, 6, and 9 in befrienders’ support related to seekers’ initial level and rate of change in mental well-being.

The findings revealed that the conditional growth model on the growth factors of befrienders’ support that predicted seekers’ psychological well-being demonstrated acceptable fit (N=100, *χ*^2^_19_=71.4; *P*<.001; RMSEA=0.06, 90% CI 0.02-0.08; SRMR=0.08; CFI=0.98; BIC=4599.26). None of the covariates for befrienders’ support and seekers’ psychological well-being were significant. The initial level of seekers’ experience of psychological symptoms positively predicted the rate of change (ie, increase) in befrienders’ support (*r*_100_=0.74; *P*<.001). Seekers with lower mental well-being at the start of the study, who reported more depressive and anxiety symptoms, experienced a greater rate of increase in support from befrienders over the course of 21 days or 3 weeks of the intervention using the Acceset platform. The rate of change or increase in befrienders’ support positively predicted the rate of change or decrease in seekers’ psychological symptoms (*r*_100_=0.22; *P*<.001). In other words, befrienders’ support that increased over the course of the intervention led to the decrease in depressive and anxiety symptoms among seekers at postintervention assessment (3 weeks) and at the follow-up assessments (6 and 9 weeks). Thus, seekers’ engagement with the digital peer support intervention over the Acceset platform demonstrated both immediate (at 3 weeks after the intervention) and prospective implications for enhanced mental well-being.

## Discussion

### Principal Findings

This RCT assessed the effectiveness of a digital peer support intervention on emerging adults’ psychological well-being—specifically, anxiety and depressive symptoms. In total, 2 aims were explored. First, we assessed the training effectiveness of the digital peer support training and intervention. Befrienders’ adoption of and fidelity to the training program was indicated by the extent to which their peer support responses demonstrated the 4 components of well-being—mattering, selfhood, compassion, and mindfulness. Hypothesis 1 was partially supported; befrienders’ peer support responses demonstrated significantly higher post- than pretraining scores for selfhood but not for the other 3 components.

Second, we examined the clinical efficacy of the digital peer support intervention on seekers’ psychological well-being. We found evidence for hypothesis 2a in that the intervention enhanced 3 components of psychological well-being among seekers over the course of the study; these were selfhood, compassion, and mindfulness. Our findings provided evidence for hypothesis 2b. Specifically, seekers in the intervention group had improved psychological well-being with lower symptoms of anxiety and depression after the intervention than seekers in the waitlist control group before the intervention. Furthermore, the effect of the intervention on seekers’ psychological well-being was sustained beyond the period of the intervention. In particular, seekers reported less psychological symptoms at postintervention assessment (3 weeks from baseline) and at the first follow-up assessment (6 weeks from baseline) but not at the second follow-up assessment (9 weeks from baseline). We also assessed seekers’ support-seeking behaviors beyond the Acceset platform and found that their perceived social support at 3, 6, and 9 weeks did not differ significantly from baseline—before the intervention.

We elucidated the mechanism of change that linked befrienders’ digital peer support to the psychological well-being of seekers. Our results lend credence to hypothesis 2c—seekers’ engagement with the digital peer support intervention demonstrated both immediate and prospective implications for their psychological well-being. The increasing trajectory of befrienders’ support over the course of the intervention predicted seekers’ experience of decrease in anxiety and depression symptoms at the postintervention (3 weeks) and follow-up (6 and 9 weeks) assessments. This study on a digital peer support intervention for emerging adult psychological well-being is the first to harness the interventional potential of 4 components of emerging adult psychological well-being and elucidate a mechanism of change involving digital peer support in enhancing psychological well-being. Our intervention also incorporated and validated digital markers of psychological well-being, especially emotion stamps, motivational Graphic Interface Formats, and functional adjustment stamps as reflecting emotionality, motivations, and psychological symptoms, respectively. Findings from our RCT provide the parameters and conditions for a novel mobile health (mHealth) peer support intervention that is effective in intervening in emerging adult psychological well-being in real-world settings.

### Effectiveness of Digital Peer Support Intervention: Training Befrienders (Aim 1)

Of the 4 components of psychological well-being, specifically, mattering, selfhood, compassion, and mindfulness, our RCT revealed that digital peer support training is effective in building emerging adults’ capacity to provide peer support responses that enhance their peers’ selfhood. This finding is consistent with an extensive body of work documenting the role of peers that is crucial in young people’s self and identity development as they establish and maintain complex social relationships, especially peer relationships [56-58]. In contrast, the lack of evidence regarding training emerging adults, specifically peer befrienders who provided support, in harnessing mattering, compassion, and mindfulness in their peer responses suggests that these components may require more deliberate efforts to be incorporated into young individuals’ support responses. Selfhood development is routine and happens in everyday life [56-58], especially during emerging adulthood, when negotiating identity exploration is a key developmental task [1], whereas compassion and mindfulness are more intentional and require active engagement of one’s cognition and insights [37-39]. Future RCTs are necessary to validate the adoption of and fidelity to training efficacy to harness the 4 components of psychological well-being in emerging adults’ online peer support responses.

### Digital Peer Support Intervention: Clinical Outcomes for Seekers and Mechanism of Change (Aim 2)

Our RCT findings on the increasing trajectories of selfhood, compassion, and mindfulness among seekers—emerging adults who sought support on the digital platform—provide empirical evidence for these 3 components as active ingredients of psychological well-being [19-21]. The characteristics of the change trajectories of selfhood, compassion, and mindfulness among seekers were aligned with the findings on befrienders’ adoption of and fidelity to the peer support training curriculum. In particular, we found that, among seekers, the moderate initial level of selfhood increased linearly throughout the course of the study, which contrasted with the low initial levels of compassion and mindfulness that increased sharply following the intervention but modestly at the follow-ups. Thus, selfhood may present greater interventional potential as an active ingredient of emerging adults’ psychological well-being—both for training peers in providing support and for those who seek support—in light of how a key developmental feature of emerging adulthood is self-exploration [1]. These findings are consistent with our ongoing meta-analytic review on how young people’s selfhood and psychopathology indicate the negative moderate relations of multiple self-variables with specific mental health conditions—anxiety and depression (PROSPERO registration: CRD42021248495).

Examining clinical efficacy, we found that the digital peer support intervention led to improved psychological well-being among emerging adults, with lower self-report symptoms of anxiety and depression at the postintervention assessment and the first follow-up assessment at 6 weeks but not at 9 weeks. Clinical outcomes associated with traditional peer support that takes place face to face are well established [28]. Importantly, our RCT provides robust empirical evidence for the clinical effectiveness of peer support delivered on a digital platform. Our ongoing systematic review and meta-analysis on peer support and mental health also found that interventional peer support has a high positive effect on mental health functioning with limited carryover effect (PROSPERO registration: CRD42022353624). Our RCT indicated that the window for harnessing the effective, therapeutic potential of online peer support for emerging adult psychological well-being via the selected peer support platform may not extend beyond 6 weeks—a finding that warrants future empirical investigation. Although we assessed and expected individuals to indicate higher perceived social support during the intervention and beyond (as compared with the level before the intervention), our findings were contrary to expectations. Perceived social support is typically conceptualized and operationalized as a regular form of peer support that one obtains from close social networks [47], which may be distinct from active engagement in and solicitation of peer support from peers with common lived experiences from the community [59].

By elucidating the mechanism of change, we found that emerging adults with lower psychological well-being at the start of the study, who reported more depressive and anxiety symptoms, experienced greater online peer support from befrienders over the course of the intervention using the Acceset platform. This result provides important insights into the therapeutic benefits of active solicitation of support from peers in the community for enhancing psychological well-being among emerging adults, especially college students [60], and lends credence to the social sharing of emotions framework [12,13]. In particular, emotional disclosure on digital platforms functions as a psychological process of support that facilitates emotion regulation and recovery and mitigates the experience of anxiety and depressive symptoms among emerging adults. More importantly, our findings revealed that online peer support increased over the course of the intervention, which predicted the pronounced decrease in emerging adults’ (seekers) psychological symptoms at the postintervention assessment (3 weeks) and the sustained level of symptoms at the follow-up assessments (6 and 9 weeks). Thus, engagement with the digital peer support intervention demonstrated both immediate and prospective implications for enhanced psychological well-being among emerging adults.

### Limitations and Conclusions

A possible limitation of this RCT of a digital peer support intervention for emerging adult psychological well-being is the use of self-report measures for assessing clinical outcomes, which could be subject to under- or overestimation of anxiety and depressive symptoms [45]. However, the use of self-report measures was aligned with the confidentiality and feasibility considerations given the anonymity of seeker-befriender-moderator interactions on the platform and is consistent with the standard procedure of peer-led mHealth interventions for young people [61]. Another limitation is the preliminary results on validating the digital markers of psychological well-being on the Acceset platform. Future research may assess additional evidence to further evaluate the actionability of these digital markers. In turn, these markers could potentially be used to triangulate evidence from self-report measures and physiological biomarkers, such as cortisol levels and blood pressure, to enhance the validity and reliability of clinical outcomes involving emerging adults’ psychological well-being [62]. Furthermore, the evidence built on the single-site superiority trial outlined in this study limits the external validity of the digital peer support intervention in real-world settings [30]. Future research should consider properly powered and rigorous studies using multiple trials of strategies and multiple sites for potential expansion into larger cohorts and potential downstream studies that could provide insight into longer-term retention to build the evidence on the effects of digital interventions in addressing emerging adult mental health.

Notwithstanding these limitations, this study’s development and validation of a novel digital innovation realize important contributions to the field of emerging adult mental health [25,30]. The key strengths of the proposed intervention are the scalability and sustainability of the digital peer support intervention. Existing evidence suggests that for scalability of web-based nonprofessional peer support training to attain the desired reach, it should incur minimal cost, not be constrained by geographical locations, and be available to individuals from diverse backgrounds and abilities [26,61]. Congruent with these findings, the studied digital peer support intervention is designed to maximize reach and is widely accessible at scale by adopting dual community engagement approaches (active and consultative) to drive optimal user engagement [30]. Our intervention uses the active method of community engagement in providing web-based safety support for young people. This form of peer support is affordable and readily available as it draws on the common lived experiences of the community, with peers functioning as befrienders and moderators who provide their peer seekers with emotional support. By using the community consultative method, college students aged 19 to 25 years from institutes of higher learning (IHLs) in Singapore were consulted in co-designing the Acceset platform. Relatedly, a unique aspect of our intervention is that it taps into community-driven cocreation, validation, and potential deployment of mHealth interventions for mental health by leveraging technology and engaging clinicians, psychologists, and counselors in the community to further the sustainability of digital peer support. This intervention may represent a scalable, sustainable, and low-cost prevention strategy that has therapeutic potential in supporting the psychological well-being of young people.

There are potential recommendations for future design and implementation of digital peer support for youth mental health based on results from our RCT and those from a systematic review and meta-analysis of RCTs on the sustainable effects of mental health interventions for students from IHLs [63-65]. First, digital peer support interventions need to maximize the effectiveness and sustainability of psychological interventions for mental health, especially for emerging adults, by emphasizing the importance of a multi-systemic approach [63-65] that entails contributions from the individual, community, and societal levels. Second, to establish and maintain the dynamics of the digital peer support platform, future interventions can consider a system comprising seekers, befrienders, and moderators. This dynamic system is characterized by individual contributions and the opportunity for seekers to join the peer support network as befrienders to expand the online support groups. Third, to normalize digital peer support as a new service delivery model, collaborations with policy makers and IHLs in designing safety standards and protocols for emerging adult engagement with digital peer support is necessary as a next step forward.

## Appendix

Multimedia Appendix 1 Coding scheme and supplementary analyses.

Multimedia Appendix 2 CONSORT-eHEALTH checklist (V 1.6.1).

## Abbreviations

BIC: Bayesian information criterion
CFI: comparative fit index
GAD-7: 7-item General Anxiety Disorder Scale
IHL: institute of higher learning
IRB: Institutional Ethics Review Board
mHealth: mobile health
NUS: National University of Singapore
PHQ-9: 9-item Patient Health Questionnaire
RCT: randomized controlled trial
RMSEA: root mean square error of approximation
SRMR: standardized root mean square residual
