# Supplementary material for: A Digital Peer Support Platform to Translate Online Peer Support for Emerging Adult Mental Well-being: Randomized Controlled Trial
Source: JMIR Ment Health. 2023 Apr 18;10:e43956. doi: 10.2196/43956 (PMC10155088; doi:10.2196/43956)
Supplement: Multimedia Appendix 1 [file mental_v10i1e43956_app1.docx]

**Supplementary Materials**

Table S1

*Coding Scheme with Samples of Participants’ Extracted Letter Changes (refer to protocol by Yeo et al., 2022 for details)*

| **Step 1: Identify and record relevant responses from participants’ letter exchanges**   - Two coders (i.e., undergraduate research assistants) were provided with examples of emotional disclosure from previous studies (e.g., Michikyan et al., 2015) - Coders manually reviewed the letter exchanges and identified materials pertinent to emotional disclosure. | **Definition of emotional disclosure :**  Interpersonal communication of significant and personal experiences with mild to strong positivity and negativity with one’s close social network (e.g., “I was exhilarated to receive a good grade on an exam” or “I was distressed being involved in a car accident”).  Seeker #1:  I recently applied for the A*star internship award along with my friend, and we both got shortlisted to attend an e-interview for the respective projects we chose. Her supervisor scheduled for the interview really fast and she managed to get the offer, while my supervisors still have not contacted me yet. This has dampened my spirits as I'm worried that I will not be able to get it. What if a lot of students chose the project I want, is that why the supervisors still have not contacted me yet? What if they decided to just take up someone already without interviewing me? I really want to intern at A*Star as I feel that it will be a great learning experience, so the thought of my friend getting in and me not is making me extremely jealous and angry. I hate how insecure I feel about myself. What if I don't ace the interview also? There's so many people that's smarter than me, more well-versed than me. I hate feeling like this. I just wish they would e-mail me soon for the interview so at least I can get one worry out of the way.  Befriender#1:  Hi! Just want to let you know that I have also been in your situation before and hope you are doing better now when you are reading this letter. Perhaps you are a little like me, slightly more competitive than the average human being, but that is totally alright! what you are feeling now is perfectly fine and only shows that you have a heart to become a better person. However, do not that stand in your way. In your case, take your friend's application aside, take the other applicants aside. How do you feel truly about yourself and the application you have submitted. That should be what defines you and your application. Meanwhile, keep yourself busy with other tasks. The last thing you would want is for this "pending outcome" to hold you back in your daily life. There is much more than this application in your life. Believe in your own abilities and do not let this internship award define you and most importantly, your confidence in your own ability! Do send me another letter if you have questions and anything at all! Best Regards |
| --- | --- |
| **Step 2: Discuss and critique the set of indicators for coding emotional experiences**   - Mattering was coded whenever the seekers’ disclosure indicated how important they were to others. For befrienders, when their support responses indicate how important seekers were. - Selfhood was coded whenever seekers’ disclosure and befrienders’ support responses indicated self knowledge, interpersonal self, and self-agency - Compassion was coded whenever seekers’ peer emotional disclosure and befrienders’ peer support responses reflected (1) an awareness of suffering, (2) affective concern, (4) a responsiveness or readiness to help relieve that suffering (motivational). - Mindfulness was coded when seekers’ peer emotional disclosure and befrienders’ peer support responses indicated flexible cognitive state in which they are actively present and notice novel aspects in both the environment and one’s perspectives | Definitions of four components of psychological well-being were provided:   1. **Mattering**—the degree to which befrienders’ responses indicated how important seekers are to others, and for seekers their importance to others using the Rosenberg Mattering Scale. 2. **Selfhood**— the extent to which befrienders’ responses built seekers’ self knowledge, interpersonal self, and self-agency using the **Rosenberg Global Self-Esteem Scale, Self-Consciousness Scale, General self-efficacy and Social self-efficacy Scales** (e.g., the peer is a person of worth, when the peer makes plans, he/she is certain that he/she can make them work, and the peer does not handle himself/herself well in social gatherings).   For seekers—the extent to which their emotional disclosure reflected self knowledge, interpersonal self, and self-agency using same subscales and items, except with a different item-stem (e.g., “he/she is a person of worth, at least on an equal plane with others” and “he/she has a number of good qualities”).  A composite selfhood score was obtained by averaging the ratings across all four selfhood scales for both befrienders and seekers.   1. **Compassion—**the extent to which befrienders’ peer support responses and seekers’ peer emotional disclosure reflected (1) an awareness of suffering, (2) affective concern, (4) a responsiveness or readiness to help relieve that suffering (motivational) using the compassion cultivation training (CCT) program. 2. **Mindfulness--**Befrienders’ support responses and seekers’ peer emotional disclosure were evaluated based on the degree to which they harness mindful self-acceptance:   (1) identify novel aspects of the situation or perspective;  (2) demonstrate “work in progress” by using possibility words, such as “could be” and offering other interpretations of the situation;  (3) highlight puzzles and paradoxes for example, how individuals may feel victimized yet is responsible for being in that situation;  (4) notice humorous aspects of the situation;  (5) perceive the situation from multiple perspectives;  (6) consider alternative (useful) aspects of a problematic context or the silver lining;  (7) emphasize a mental file of positive memories; (8) encourage mindfulness journaling.  For assessing mattering, selfhood, compassion and mindfulness, a 4-point scale ranging from 1 (not at all) to 4 (a lot) was used by raters. |
| **Step 3: Apply the coding scheme to a sample of responses (approximately 20%) and refine it based on this initial coding** | Samples from participants’ letter exchanges that were extracted (N = 192):  **Mattering**  Seeker#2:  I study alone at home most of the time because I tend to end up talking with my friends if we are studying together :')  Befriender#2  For me, what helped me cope with the situation was to talk to friends who I trust and let out the negative emotions within me through these conversations.. Hope the advice helped, and I am always here with you till you feel better about the situation :)  **Selfhood**  Seeker#3  I really want to intern at A*Star as I feel that it will be a great learning experience, so the thought of my friend getting in and me not is making me extremely jealous and angry. I hate how insecure I feel about myself. What if I don't ace the interview also? There's so many people that's smarter than me, more well-versed than me. I hate feeling like this.  Befriender#3  It is important for you to prioritise yourself and find other motivations in life. It can be a hobby, goals you are working towards, or even just spending more time with your family  **Compassion**  Seeker#4  I feel pressurised by the overwhelming workload in university. I have been having back to back lessons and around 3 examinations a week, with multiple project deadlines to meet. On top of the heavy workload, I am also taking a language module which requires consistent revision and practice. Hence, I find it hard to strike a balance between school work and things I enjoy.  Befriender#4  Firstly, it is perfectly normal for you to be feeling how you are feeling now. It probably wasn't easy for you to muster the courage to confess to your friend. I just want to let you know that it is normal to take some time to figure it all out, and you are not alone in the journey as I will be here for you as well.  **Mindfulness**  Seeker#4  Hello and yep I do like the Japanese language module even though it requires a lot of commitment and time. I have never regretted taking up the challenge of learning a new language. Most of the times i would end up talking in libraries too or even worse, accidentally laughing loudly, much to the dismay of other strangers around me who are trying to study D:  Befriender#4  The pandemic has definitely created problems when it comes to connecting with our loved ones and I'm sure there are people out there who can relate to what you're feeling, i.e. the disconnectedness and perhaps even loneliness. Remember that the depth of relationships is not usually dependent on the physical connection but mainly on the emotional connection we have with the other party. I agree that meeting online doesn't have the same feeling as meeting up in person, but you can still connect with people online and have that mental and emotional connection. |
| **Step 4: Re-evaluate the dimensions in the coding scheme to ensure mutual exclusiveness and exhaustiveness** | Coders discussed with the lead author on the relevance of the different subscales in the coding scheme for assessing the four components of psychological well-being:  a. Mattering—Rosenberg Mattering Scale  b. Selfhood—**Rosenberg Global Self-Esteem Scale, Self-Consciousness Scale, General self-efficacy and Social self-efficacy Scales.**  **c. Compassion—**compassion cultivation training (CCT) program.  d. Mindfulness—Mindfulness and self-acceptance Scale |
| **Step 5: Pre-test the coding scheme with different coders (two other undergraduate research volunteers)** | Definitions of four components of psychological well-being were provided:   1. Mattering 2. Selfhood 3. Compassion 4. Mindfulness |
| **Step 6:**  **Finalize the coding scheme with the agreement of the coders.** | After discussion with the two undergraduate research volunteers, the above coding scheme was finalized.  20% of all the letter exchanges were double coded to ensure continued reliability. Specifically, the lead author trained single coder (undergraduate research assistants) to code each letter exchange, and check in with them to avoid coder drift during coding process.  Then, the two coders applied the coding scheme to the remaining letter exchanges. The lead author and the two coders reconciled differences in coding through discussions. |

Table S2

| Time point |  | Goodness of fit | | | | | | |
| --- | --- | --- | --- | --- | --- | --- | --- | --- |
|  | Model | RMSEA | 90% CI | CFI | SRMR | BIC | *χ*^2^ | df |
| Befrienders’ Support |  |  |  |  |  |  |  |  |
| T_1_ | **One-factor** | **0.05** | **[0.04, 0.07]** | **0.97** | **0.03** | **1483.58** | 468.34**^**^** | **230** |
|  | Four-factor | 0.14 | [0.15, 0.20] | 0.59 | 0.14 | **1831.00** | **608.85**^**^ | 224 |
| T_2_ | **One-factor** | **0.01** | **[0.01, 0.05]** | **0.91** | **0.07** | **1612.86** | **415.68^**^** | **230** |
|  | Four-factor | 0.22 | [0.19, 0.24] | 0.34 | 0.18 | 1673.37 | 497.52^**^ | 224 |
| T_3_ | **One-factor** | **0.07** | **[0.06, 0.09]** | **0.98** | **0.04** | **1209.14** | **417.31^**^** | **230** |
|  | Four-factor | 0.12 | [0.11, 0.14] | 0.65 | 0.16 | 1306.64 | 466.22^**^ | 224 |
| T_4_ | **One-factor** | **0.04** | **[0.02, 0.04]** | **0.98** | **0.08** | **1615.60** | **535.60^**^** | **230** |
|  | Four-factor | 0.20 | [0.19, 0.22] | 0.59 | 0.10 | 1833.86 | 761.86*^*^ | 224 |
| Seekers’ Well-Being |  |  |  |  |  |  |  |  |
| T_1_ | **One-factor** | **0.07** | **[0.05, 0.09]** | **0.91** | **0.07** | **2986.32** | **160.01^**^** | **104** |
|  | Two-factor | 0.11 | [0.09, 0.13] | 0.80 | 0.10 | 3056.76 | 235.06^**^ | 103 |
| T_2_ | **One-factor** | **0.06** | **[0.01, 0.09]** | **0.94** | **0.06** | **1956.25** | **288.77^**^** | **104** |
|  | Two-factor | 0.18 | [0.16, 0.20] | 0.71 | 0.10 | 1981.84 | 318.50^**^ | 103 |
| T_3_ | **One-factor** | **0.02** | **[0.00, 0.04]** | **0.95** | **0.08** | **1479.91** | **184.74^**^** | **104** |
|  | Two-factor | 0.13 | [0.11, 0.16] | 0.82 | 0.09 | 1495.33 | 204.15^**^ | 103 |
| T_4_ | **One-factor** | **0.04** | **[0.01, 0.08]** | **0.93** | **0.08** | **1158.42** | **201.57^**^** | **104** |
|  | Two-factor | 0.17 | [0.14, 0.02] | 0.77 | 0.09 | 1191.53 | 238.44^**^ | 103 |

*Measurement Models of Befrienders’ Support and Seekers’ Psychological Well-Being: Fit Indices*

*Note.* RMSEA = root mean square error of approximation; CI = confidence interval; CFI = comparative fit index; SRMR = standardized root mean square residual; BIC = Bayesian Information Criterion; df = degrees of freedom. The one-factor model indicated a better fit that the four-factor model. All factor loadings are standardized. T_1_=prior to intervention (0 week), T_2_=post intervention (3 weeks), T_3_=first follow-up (6 weeks), T_4_=second follow-up (9 weeks)

^*^*p* < .05. ^**^*p* < .01

| Table S3  *Correlations and Descriptive Statistics for Support and Psychological Well-Being Among Seekers Across Time* | | | | | | | | | | |
| --- | --- | --- | --- | --- | --- | --- | --- | --- | --- | --- |
|  | 1. | 2. | 3. | 4. | 9. | 10. | 11. | 12. | M | SD |
| 1. PSS_1_ | - |  |  |  |  |  |  |  | 63.38 | 9.78 |
| 2. PSS_2_ | .61 | - |  |  |  |  |  |  | 63.92 | 7.32 |
| 3. PSS_3_ | .32 | .60 | - |  |  |  |  |  | 62.25 | 9.53 |
| 4. PSS_4_ | .31 | .60 | .79 | - |  |  |  |  | 62.60 | 11.30 |
| 1. 9. PWB_1_ | .13 | .29 | .23 | .19 | - |  |  |  | 12.06 | 3.57 |
| 1. 10. PWB_2_ | .21 | .39 | .28 | .19 | .52 | - |  |  | 9.49 | 2.67 |
| 1. 11. PWB_3_ | .20 | .32 | .26 | .16 | .54 | .76 | - |  | 8.45 | 2.69 |
| 1. 12. PWB_4_ | .36 | .19 | .18 | .18 | .52 | .69 | .78 | - | 10.26 | 3.57 |
| *Note.* PSS = Perceived Social Support; PWB = Psychological Well-Being; M = Mean; SD = Standard Deviation; _1_ = first-time point (prior to intervention, 0 week); _2_ = second-time point (post intervention, 3 weeks); _3_ = third-time point (first follow up, 6 weeks); _4_ = fourth-time point (second follow up, 9 weeks).  *^*^p* < .05; ^**^ *p* < .01. | | | | | | | | | | |

**Supplementary analyses on implementation outcomes—feasibility and acceptability of the intervention**

**Research Question 1:** Is the digital peer support intervention (i.e., the platform) feasible and acceptable (i.e., safe and timely) in sustaining access to online peer support for seekers and befrienders?

**Hypothesis 1:** The digital peer support intervention is feasible and acceptable in offering an ongoing mechanism of support for emerging adults in two ways:

(i) The initial and sustained engagement of seekers-befrienders-moderators’ interactions—this is indexed by the waiting period for seekers to receive a response from befrienders and moderators across 4 time points: baseline (prior to intervention), 3 weeks (conclusion of intervention), 6 and 9 weeks (carry-over effect assessment). Delays were defined as >48 hours waiting period and were categorized based on technical and human factors (e.g., the befriender is not able to log into the platform to respond in time, and befriender drops out from the study and no replacement is found in time, respectively). An acceptable response time was defined as being within 48 hours of initial contact, which was emphasized during Acceset digital peer support training for befrienders.

(ii) The use of technical features of the Acceset platform—this was measured by the seeker drop-out rate in engaging in the Acceset platform, the number of visits on the study registration website, the number of participants registered with Acceset, the average number of letters exchanged, and the average number of emotion and functional adjustment stickers and motivation GIF used.

**Research Question 2**: Is the digital peer support intervention (i.e., the platform) feasible and acceptable (i.e., safe and timely) as an ongoing mechanism that identifies individuals who have an unacceptably high risk for mental health conditions?

**Hypothesis 2**: The digital peer support intervention is feasible and acceptable in identifying individuals who have an unacceptably high risk for mental health conditions related to depression and suicidality before and during the intervention based on three means. Accordingly, the Acceset algorithm detected the content of letters, moderators vetted the content of seekers’ letters, and seekers provided responses on the 9-item Patient Health Questionnaire (PHQ-9; [1], refer to implementation outcomes for details).

**Results**

With respect to RQ1, H1, RQ2 and H2, we evaluated the feasibility and acceptability of sustaining access to online peer support in terms of: (i) initial and sustained engagement of seekers, befrienders and moderators (i.e., certified counsellor), (ii) the use of technical features of the Acceset platform, and (iii) the identification of participants with high risk for mental health conditions relating to depression and suicidality. The engagement of seeker-befriender-moderator interactions across the 4 time points, including baseline (prior to intervention), 3 weeks (conclusion of intervention), 6 and 9 weeks (carry-over effect assessment), was indexed by waiting period for seekers to receive a response. On average, the waiting period for seekers to receive a response was 10.27 hours (SD = 7.61), which was within 48 hours—the acceptable response period. Thus, there were no apparent delays, which were defined by the IRB protocol as > 48 hours of waiting period. To ascertain the reasons for the waiting period of 10.72 hours, we found that it was related primarily to human factors. In particular, the study trial was conducted during the academic semester for participants, who are college students with individualized and varying timetables which affected when and how befrienders (and seekers) were able to log onto the Acceset platform. Throughout the three week intervention, there was only one instance where a befriender reported a technical issue of not being able to access the Acceset platform and this was resolved within one hour.

With regards to evaluating the sustainability of the digital peer support platform and process, the use of technical features of the Acceset platform demonstrated the feasibility and acceptability of continued access to online peer support. Specifically, the seeker drop-out rate (from both the intervention and waitlist control arms) was 4% (4 out of 100 seekers) throughout the 3-week engagement with the Acceset intervention. In total, seekers, befrienders and moderators made a total of 653 visits on the study registration website and log-on events to the platform. In total, 104 seekers, 37 befrienders, and 2 moderators registered with Acceset for the study. A total of 192 letters were exchanged between seekers and befrienders (1 exchange comprised a letter that a seeker sent and a reply from a befriender) during the 3-week intervention for both the intervention and waitlist control arms. On average, 4.67 letters were exchanged per day for 21 days (3 weeks). More than half of the letter content were related to interpersonal relationships (friends, family and romantic partners; 57%) and the remaining pertained to health (22%), school and future goals (21%). On average, 6.21 emotion and functional adjustment stickers and 3.62 motivation GIFs, which are digital features on Acceset platform, were used per day during the 21 days of letter exchange/intervention.

We also evaluated if sustained access to online peer support was feasible and acceptable in identifying participants with high risk for mental health conditions related to depression and suicidality, and referring them to appropriate mental health support providers before and during the course of the study. At recruitment, we assessed the percentage of participants identified as being at an unacceptably high risk for depression and suicidality (i.e., meeting clinical cut-off of PHQ-9 > 9) and excluded them from the study. Our assessment found that seekers’ (Mean = 5.89, SD = 3.69, range = 0 to 9) and befrienders’ (Mean = 3.31, SD = 2.59, range = 0 to 8) depression score was below the cut-off at recruitment assessment (i.e., baseline prior to intervention). Thus, none of them were excluded from the study. During the course of the study, seekers’ self-report at 3 weeks (conclusion of intervention) found that 9 of them had PHQ-9 > 9. 8 and 6 of them had scores above the clinical cut-off at 6 and 9 weeks (carry-over effect assessment), respectively. For befrienders, none of them met the clinical cut-off at 3, 6, and 9 weeks.

Seekers’ self-report responses on the PHQ-9 functioned as the primary risk assessment for mental health conditions relating to depression and suicidality that was used to make referral to appropriate mental health support providers. These seekers were referred to appropriate mental health providers including counselling centers and hotlines within the NUS campus during the course of the study. Additionally, there were two secondary means of identifying participants with high risk for depression and suicidality. First, the Acceset algorithm scanned the letter exchanges for high negativity and suicidal thoughts and depression. Importantly, the algorithm identified the same seekers whose self-reported PHQ-9 scores were above the clinical cut-off. Second, moderators and seekers used the PHQ-9 as the systematized risk assessment tool in identifying letter exchanges with high negativity and suicidal thoughts and expression. Of the 192 letter exchanges throughout the 21 days of intervention, 72 (37.5%) of them were correctly identified by befrienders as indicating an unacceptably high risk for depression and suicidality, and they were referred to and confirmed by moderators. We found that the three means of identifying seekers with high risk for mental health conditions triangulated. In other words, seekers who met clinical cut-off with self-reported PHQ-9 scores had letter exchanges that were detected by the Acceset algorithm and identified by befrienders-moderators through their risk assessments. The study team informed the counselling center on the NUS campus and referred at-risk seekers for appropriate and timely support throughout the course of the intervention, as well as at week 6 and 9 follow up assessments on all seekers (to measure carryover effects) to ensure their psychological safety. As research mental health intervention has noted, confidentiality can circumscribe post-referral processing and follow-up of referred participants as they might not be receptive or responsive to the referral [2].

**Discussion**

Our findings supported H1 on the feasibility and acceptability of the digital peer support intervention in providing an ongoing mechanism of support. There was initial and sustained engagement of seeker-befriender-moderator interactions, and seekers’ use of digital features of psychological well-being that involved emotionality (i.e., positivity and negativity), motivations, and functional adjustment (i.e., internalizing and externalizing behaviors) on the Acceset platform. Our findings also supported H1c on the feasibility and acceptability of the intervention in identifying seekers with high risk for suicide and depression.

Our evaluation of the implementation outcomes of the digital peer support platform via Acceset demonstrated feasibility and acceptability of online peer support for emerging adults, and importantly, provided information on the parameters for real-world implementation [3, 4]. We found both initial and sustained engagement of the platform among emerging adults, with peer befrienders providing timely support (i.e., without delays of more than 48 hours). On average, befrienders spent 10.27 hours on the platform responding to peers who sought support (i.e., seekers). The low drop out rate (4%), with 4 to 5 letter exchanges and the use of 3 to 7 digital features comprising emotion and functional adjustment stickers and 3.62 motivation GIF per day for 21 days of intervention provide converging evidence for designing features of digital platform as markers of emerging adults’ psychological well-being and the utility of peer emotional disclosure process [5]. These findings addressed the lacunae in our current understanding of and burgeoning interests among education stakeholders, mental health professionals, and policy makers in harnessing the therapeutic potential of peer support on digital platforms in enhancing young people psychological, especially emerging adults who experienced heightened risk for anxiety and depression [6, 7].

A critical aspect of designing and implementing peer support on digital platforms is to provide evidence for its feasibility and acceptability of identifying young people with high risk of mental health conditions involving depression and suicidality and referring them to mental health professionals [8, 9]. Findings from our RCT intervention on digital peer support underscore three means in building the capacity of digital platforms and triangulating evidence to distinguish high risk individuals and ensure their psychological safety. Accordingly, PHQ-9 can be used as the systematized risk assessment tool for emerging adults seeking support and peers providing support on the digital platforms [10], as well as leveraging artificial intelligence technology by incorporating an algorithm on the platform that detects peer emotional disclosure for high negativity and expressions of suicidal thoughts and depression [11]. Equally important for digital peer support intervention is to establish connections with appropriate mental health providers including counselling centers and hotlines to provide high risk individuals with referrals to mental health professionals [12].

**References**

1. Kroenke K, Spitzer RL, Williams JB. The PHQ‐9: validity of a brief depression severity measure. Journal of General Internal Medicine 2001 Sep; 16(9):606-13. [doi: 10.1046/j.1525-1497.2001.016009606.x]
2. Van Liew JR. Balancing confidentiality and collaboration within multidisciplinary health care teams. Journal of Clinical Psychology in Medical Settings 2012 Dec;19(4):411-7. [doi: 10.1007/s10880-012-9333-0]
3. Bernecker SL, Williams JJ, Caporale-Berkowitz NA, Wasil AR, Constantino MJ. Nonprofessional peer support to improve mental health: randomized trial of a scalable web-based peer counseling course. Journal of Medical Internet Research 2020; 22(9):e17164. [doi: 10.2196/17164]
4. Myers AL, Collins-Pisano C, Ferron JC, Fortuna KL. Feasibility and preliminary effectiveness of a peer-developed and virtually delivered community mental health training program (emotional CPR): pre-post study. Journal of Participatory Medicine 2021 Mar 4; 13(1):e25867. [doi: 10.2196/25867]
5. Andalibi N, Flood MK. Considerations in designing digital peer support for mental health: interview study among users of a digital support system (Buddy Project). JMIR Mental Health 2021 Jan 4; 8(1):e21819. [doi: 10.2196/21819]
6. Kaess M, Moessner M, Koenig J, Lustig S, Bonnet S, Becker K, Eschenbeck H, Rummel‐Kluge C, Thomasius R, Bauer S, ProHEAD Consortium. Editorial Perspective: A plea for the sustained implementation of digital interventions for young people with mental health problems in the light of the COVID-19 pandemic. Journal of Child Psychology and Psychiatry 2021 Jul;62(7):916-8.31. [doi: 10.1111/jcpp.13317]
7. Fortuna KL, Naslund JA, LaCroix JM, Bianco CL, Brooks JM, Zisman-Ilani Y et al. Digital peer support mental health interventions for people with a lived experience of a serious mental illness: systematic review. JMIR Mental Health 2020; 7(4):e16460. [doi: 10.2196/16460]
8. Ali K, Farrer L, Gulliver A, Griffiths KM. Online peer-to-peer support for young people with mental health problems: a systematic review. JMIR Mental Health 2015 May 19; 2(2):e4418. [doi: 10.2196/mental.4418]
9. Lattie EG, Adkins EC, Winquist N, Stiles-Shields C, Wafford QE, Graham AK. Digital mental health interventions for depression, anxiety, and enhancement of psychological well-being among college students: systematic review. Journal of Medical Internet Research 2019; 21(7):e12869. [doi: 10.2196/12869]
10. Moir F, Henning M, Hassed C, Moyes SA, Elley CR. A peer-support and mindfulness program to improve the mental health of medical students. Teaching and learning in medicine 2016 Jul 2;28(3):293-302. [doi: 10.1080/10401334.2016.1153475]
11. Roy A, Nikolitch K, McGinn R, Jinah S, Klement W, Kaminsky ZA. A machine learning approach predicts future risk to suicidal ideation from social media data. NPJ digital medicine 2020 May 26;3(1):1-2. [doi: 10.1038/s41746-020-0287-6]
12. Lee YY, Ang S, Chua HC, Subramaniam M. Peer support in mental health: a growing movement in Singapore. Ann Acad Med Singapore. 2019 Mar 1;48(3):95-7.
