# Supplementary material for: A Digital Peer Support Platform to Translate Online Peer Support for Emerging Adult Mental Well-being: Randomized Controlled Trial
Source: JMIR Ment Health. 2023 Apr 18;10:e43956. doi: 10.2196/43956 (PMC10155088; doi:10.2196/43956)
Supplement: Multimedia Appendix 2 [file mental_v10i1e43956_app2.pdf]

CONSORT-EHEALTH (V 1.6.1) - Submission/Publication Form

The CONSORT-EHEALTH checklist is intended for authors of randomized trials evaluating web-based and Internet-based applications/interventions, including mobile interventions, electronic games (incl multiplayer games), social media, certain telehealth applications, and other interactive and/or networked electronic applications. Some of the items (e.g. all subitems under item 5 - description of the intervention) may also be applicable for other study designs.

The goal of the CONSORT EHEALTH checklist and guideline is to be
a) a guide for reporting for authors of RCTs,
b) to form a basis for appraisal of an ehealth trial (in terms of validity)

CONSORT-EHEALTH items/subitems are MANDATORY reporting items for studies published in the Journal of Medical Internet Research and other journals / scientific societies endorsing the checklist.

Items numbered 1., 2., 3., 4a., 4b etc are original CONSORT or CONSORT-NPT (non-pharmacologic treatment) items.
Items with Roman numerals (i., ii, iii, iv etc.) are CONSORT-EHEALTH extensions/clarifications.

As the CONSORT-EHEALTH checklist is still considered in a formative stage, we would ask that you also RATE ON A SCALE OF 1-5 how important/useful you feel each item is FOR THE PURPOSE OF THE CHECKLIST and reporting guideline (optional).

Mandatory reporting items are marked with a red *.
In the textboxes, either copy & paste the relevant sections from your manuscript into this form - please include any quotes from your manuscript in QUOTATION MARKS,
or answer directly by providing additional information not in the manuscript, or elaborating on why the item was not relevant for this study.

YOUR ANSWERS WILL BE PUBLISHED AS A SUPPLEMENTARY FILE TO YOUR PUBLICATION IN JMIR AND ARE CONSIDERED PART OF YOUR PUBLICATION (IF ACCEPTED).
Please fill in these questions diligently. Information will not be copyedited, so please use proper spelling and grammar, use correct capitalization, and avoid abbreviations.

DO NOT FORGET TO SAVE AS PDF _AND_ CLICK THE SUBMIT BUTTON SO YOUR ANSWERS ARE IN OUR DATABASE !!!

Your response is too large. Try shortening some answers.

Eysenbach G, CONSORT-EHEALTH Group

CONSORT-EHEALTH: Improving and Standardizing Evaluation Reports of Web-based and Mobile Health Interventions
J Med Internet Res 2011;13(4):e126
URL: http://www.jmir.org/2011/4/e126/
doi: 10.2196/jmir.1923
PMID: 22209829

geckhongyeo@gmail.com (not shared) Switch account

* Required

Your name *

First Last

GeckHong

Primary Affiliation (short), City, Country *

University of Toronto, Toronto, Canada

1N.1 Institute for Health (N.1), National Univer

Your e-mail address *

abc@gmail.com

geckhongyeo@gmail.com

Your response is too large. Try shortening some answers.

Title of your manuscript *

Provide the (draft) title of your manuscript.

A Randomized Controlled Trial for a Digital Peer Support Platform to Translate Online Peer Support for Emerging Adult Mental Well-being

Name of your App/Software/Intervention *

If there is a short and a long/alternate name, write the short name first and add the long name in brackets.

Digital Peer Support

Evaluated Version (if any)

e.g. "V1", "Release 2017-03-01", "Version 2.0.27913"

not applicable

Language(s) *

What language is the intervention/app in? If multiple languages are available, separate by comma (e.g. "English, French")

English

URL of your Intervention Website or App

e.g. a direct link to the mobile app on app in appstore (itunes, Google Play), or URL of the website. If the intervention is a DVD or hardware, you can also link to an Amazon page.

Your response is too large. Try shortening some answers.

URL of an image/screenshot (optional)

https://nus.acceset.com/

Accessibility *

Can an enduser access the intervention presently?

- ○ access is free and open
- ◉ access only for special usergroups, not open
- ○ access is open to everyone, but requires payment/subscription/in-app purchases
- ○ app/intervention no longer accessible
- ○ Other:

Primary Medical Indication/Disease/Condition *

e.g. "Stress", "Diabetes", or define the target group in brackets after the condition, e.g. "Autism (Parents of children with)", "Alzheimers (Informal Caregivers of)"

Mental Well-Being (emerging adults)

Primary Outcomes measured in trial *

comma-separated list of primary outcomes reported in the trial

Four Components of Psychological Well-Being

Your response is too large. Try shortening some answers.

Secondary/other outcomes

Are there any other outcomes the intervention is expected to affect?

Perceived Social Support; Implementation Outcomes (feasibility, acceptability)

Recommended "Dose" *

What do the instructions for users say on how often the app should be used?

- (x) Approximately Daily
- ( ) Approximately Weekly
- ( ) Approximately Monthly
- ( ) Approximately Yearly
- ( ) "as needed"
- ( ) Other:

Your response is too large. Try shortening some answers.

Approx. Percentage of Users (starters) still using the app as recommended after 3 months *

- (selected) unknown / not evaluated
- 0-10%
- 11-20%
- 21-30%
- 31-40%
- 41-50%
- 51-60%
- 61-70%
- 71%-80%
- 81-90%
- 91-100%
- Other:

Overall, was the app/intervention effective? *

- (selected) yes: all primary outcomes were significantly better in intervention group vs control
- partly: SOME primary outcomes were significantly better in intervention group vs control
- no statistically significant difference between control and intervention
- potentially harmful: control was significantly better than intervention in one or more outcomes
- inconclusive: more research is needed

Your response is too large. Try shortening some answers.

Article Preparation Status/Stage *

At which stage in your article preparation are you currently (at the time you fill in this form)

- ( ) not submitted yet - in early draft status
- ( ) not submitted yet - in late draft status, just before submission
- (•) submitted to a journal but not reviewed yet
- ( ) submitted to a journal and after receiving initial reviewer comments
- ( ) submitted to a journal and accepted, but not published yet
- ( ) published
- ( ) Other:

Journal *

If you already know where you will submit this paper (or if it is already submitted), please provide the journal name (if it is not JMIR, provide the journal name under "other")

- ( ) not submitted yet / unclear where I will submit this
- ( ) Journal of Medical Internet Research (JMIR)
- ( ) JMIR mHealth and UHealth
- ( ) JMIR Serious Games
- (•) JMIR Mental Health
- ( ) JMIR Public Health
- ( ) JMIR Formative Research
- ( ) Other JMIR sister journal
- ( ) Other:

Your response is too large. Try shortening some answers.

Is this a full powered effectiveness trial or a pilot/feasibility trial? *

- ○ Pilot/feasibility
- ◉ Fully powered

Manuscript tracking number *

If this is a JMIR submission, please provide the manuscript tracking number under "other" (The ms tracking number can be found in the submission acknowledgement email, or when you login as author in JMIR. If the paper is already published in JMIR, then the ms tracking number is the four-digit number at the end of the DOI, to be found at the bottom of each published article in JMIR)

- ○ no ms number (yet) / not (yet) submitted to / published in JMIR
- ◉ Other: JMH ms#43956

TITLE AND ABSTRACT

1a) TITLE: Identification as a randomized trial in the title

1a) Does your paper address CONSORT item 1a? *

I.e does the title contain the phrase "Randomized Controlled Trial"? (if not, explain the reason under "other")

- ◉ yes
- ○ Other:

Your response is too large. Try shortening some answers.

1a-i) Identify the mode of delivery in the title

Identify the mode of delivery. Preferably use “web-based” and/or “mobile” and/or “electronic game” in the title. Avoid ambiguous terms like “online”, “virtual”, “interactive”. Use “Internet-based” only if Intervention includes non-web-based Internet components (e.g. email), use “computer-based” or “electronic” only if offline products are used. Use “virtual” only in the context of “virtual reality” (3-D worlds). Use “online” only in the context of “online support groups”. Complement or substitute product names with broader terms for the class of products (such as “mobile” or “smart phone” instead of “iphone”), especially if the application runs on different platforms.

subitem not at all important

- 1 ○
- 2 ○
- 3 ○
- 4 ○
- 5 ◉

essential

Clear selection

Does your paper address subitem 1a-i? *

Copy and paste relevant sections from manuscript title (include quotes in quotation marks "like this" to indicate direct quotes from your manuscript), or elaborate on this item by providing additional information not in the ms, or briefly explain why the item is not applicable/relevant for your study

"A Randomized Controlled Trial for a Digital Peer Support Platform to Translate Online Peer Support for Emerging Adult Mental Well-being"

Your response is too large. Try shortening some answers.

1a-ii) Non-web-based components or important co-interventions in title

Mention non-web-based components or important co-interventions in title, if any (e.g., “with telephone support”).

subitem not at all important

1 (selected)

2

3

4

5

essential

Clear selection

Does your paper address subitem 1a-ii?

Copy and paste relevant sections from manuscript title (include quotes in quotation marks "like this" to indicate direct quotes from your manuscript), or elaborate on this item by providing additional information not in the ms, or briefly explain why the item is not applicable/relevant for your study

Not applicable. No non-web-based components

Your response is too large. Try shortening some answers.

1a-iii) Primary condition or target group in the title

Mention primary condition or target group in the title, if any (e.g., “for children with Type I Diabetes”) Example: A Web-based and Mobile Intervention with Telephone Support for Children with Type I Diabetes: Randomized Controlled Trial

subitem not at all important

1 ○

2 ○

3 ○

4 ○

5 ◉

essential

Clear selection

Does your paper address subitem 1a-iii? *

Copy and paste relevant sections from manuscript title (include quotes in quotation marks "like this" to indicate direct quotes from your manuscript), or elaborate on this item by providing additional information not in the ms, or briefly explain why the item is not applicable/relevant for your study

"for Emerging Adult Mental Well-being"

1b) ABSTRACT: Structured summary of trial design, methods, results, and conclusions

NPT extension: Description of experimental treatment, comparator, care providers, centers, and blinding status.

Your response is too large. Try shortening some answers.

1b-i) Key features/functionalities/components of the intervention and comparator in the METHODS section of the ABSTRACT

Mention key features/functionalities/components of the intervention and comparator in the abstract. If possible, also mention theories and principles used for designing the site. Keep in mind the needs of systematic reviewers and indexers by including important synonyms. (Note: Only report in the abstract what the main paper is reporting. If this information is missing from the main body of text, consider adding it)

subitem not at all important

- 1 (selected)
- 2
- 3
- 4
- 5

essential

Clear selection

Does your paper address subitem 1b-i? *

Copy and paste relevant sections from the manuscript abstract (include quotes in quotation marks "like this" to indicate direct quotes from your manuscript), or elaborate on this item by providing additional information not in the ms, or briefly explain why the item is not applicable/relevant for your study

Information about key features/functionalities/components of the intervention and comparator are in the published protocol (Yeo G, Chang W, Lee LN, Oon M, Ho D. A Digital Peer Support Platform to Translate Web-Based Peer Support for Emerging Adult Mental Well-being: Protocol for a Randomized Controlled Trial. JMIR research protocols 2022 Sep 20;11(9):e34602. [doi: 10.2196/34602)

Your response is too large. Try shortening some answers.

1b-ii) Level of human involvement in the METHODS section of the ABSTRACT

Clarify the level of human involvement in the abstract, e.g., use phrases like "fully automated" vs. "therapist/nurse/care provider/physician-assisted" (mention number and expertise of providers involved, if any). (Note: Only report in the abstract what the main paper is reporting. If this information is missing from the main body of text, consider adding it)

subitem not at all important

- 1 (selected)
- 2
- 3
- 4
- 5

essential

Clear selection

Does your paper address subitem 1b-ii?

Copy and paste relevant sections from the manuscript abstract (include quotes in quotation marks "like this" to indicate direct quotes from your manuscript), or elaborate on this item by providing additional information not in the ms, or briefly explain why the item is not applicable/relevant for your study

Information about the level of human involvement are in the published protocol (Yeo G, Chang W, Lee LN, Oon M, Ho D. A Digital Peer Support Platform to Translate Web-Based Peer Support for Emerging Adult Mental Well-being: Protocol for a Randomized Controlled Trial. JMIR research protocols 2022 Sep 20;11(9):e34602. [doi: 10.2196/34602)

Your response is too large. Try shortening some answers.

1b-iii) Open vs. closed, web-based (self-assessment) vs. face-to-face assessments in the METHODS section of the ABSTRACT

Mention how participants were recruited (online vs. offline), e.g., from an open access website or from a clinic or a closed online user group (closed usergroup trial), and clarify if this was a purely web-based trial, or there were face-to-face components (as part of the intervention or for assessment). Clearly say if outcomes were self-assessed through questionnaires (as common in web-based trials). Note: In traditional offline trials, an open trial (open-label trial) is a type of clinical trial in which both the researchers and participants know which treatment is being administered. To avoid confusion, use "blinded" or "unblinded" to indicated the level of blinding instead of "open", as "open" in web-based trials usually refers to "open access" (i.e. participants can self-enrol). (Note: Only report in the abstract what the main paper is reporting. If this information is missing from the main body of text, consider adding it)

subitem not at all important

1 (selected)

2

3

4

5

essential

Clear selection

Does your paper address subitem 1b-iii?

Copy and paste relevant sections from the manuscript abstract (include quotes in quotation marks "like this" to indicate direct quotes from your manuscript), or elaborate on this item by providing additional information not in the ms, or briefly explain why the item is not applicable/relevant for your study

Information about the level of human involvement are in the published protocol (Yeo G, Chang W, Lee LN, Oon M, Ho D. A Digital Peer Support Platform to Translate Web-Based Peer Support for Emerging Adult Mental Well-being: Protocol for a Randomized Controlled Trial. [illegible]

Your response is too large. Try shortening some answers.

1b-iv) RESULTS section in abstract must contain use data

Report number of participants enrolled/assessed in each group, the use/uptake of the intervention (e.g., attrition/adherence metrics, use over time, number of logins etc.), in addition to primary/secondary outcomes. (Note: Only report in the abstract what the main paper is reporting. If this information is missing from the main body of text, consider adding it)

subitem not at all important

1 ○

2 ○

3 ○

4 ◉

5 ○

essential

Clear selection

Does your paper address subitem 1b-iv?

Copy and paste relevant sections from the manuscript abstract (include quotes in quotation marks "like this" to indicate direct quotes from your manuscript), or elaborate on this item by providing additional information not in the ms, or briefly explain why the item is not applicable/relevant for your study

"The RCT found implementation effectiveness of digital peer support training—specifically, befrienders' peer support responses demonstrating significantly higher post- than pre-training scores in selfhood. The digital peer support intervention indicated feasibility and acceptability as an ongoing mechanism of support. The intervention demonstrated clinical effectiveness in enhancing selfhood, compassion and mindfulness, and in lowering depressive and anxiety symptoms among seekers in the intervention group at post intervention than seekers in the waitlist control group prior to intervention. The effect of the intervention on seekers' psychological well-being was sustained beyond the period of the intervention. The mechanism of change revealed that seekers' engagement of the intervention had both immediate and prospective implications for their psychological well-being."

Your response is too large. Try shortening some answers.

1b-v) CONCLUSIONS/DISCUSSION in abstract for negative trials

Conclusions/Discussions in abstract for negative trials: Discuss the primary outcome - if the trial is negative (primary outcome not changed), and the intervention was not used, discuss whether negative results are attributable to lack of uptake and discuss reasons. (Note: Only report in the abstract what the main paper is reporting. If this information is missing from the main body of text, consider adding it)

subitem not at all important

- (●) 1
- ( ) 2
- ( ) 3
- ( ) 4
- ( ) 5

essential

Clear selection

Does your paper address subitem 1b-v?

Copy and paste relevant sections from the manuscript abstract (include quotes in quotation marks "like this" to indicate direct quotes from your manuscript), or elaborate on this item by providing additional information not in the ms, or briefly explain why the item is not applicable/relevant for your study

The trial outcome was positive (primary outcome was changed)

INTRODUCTION

2a) In INTRODUCTION: Scientific background and explanation of rationale

Your response is too large. Try shortening some answers.

2a-i) Problem and the type of system/solution

Describe the problem and the type of system/solution that is object of the study: intended as stand-alone intervention vs. incorporated in broader health care program? Intended for a particular patient population? Goals of the intervention, e.g., being more cost-effective to other interventions, replace or complement other solutions? (Note: Details about the intervention are provided in “Methods” under 5)

subitem not at all important

1 ○

2 ○

3 ○

4 ○

5 ◉

essential

Clear selection

Does your paper address subitem 2a-i? *

Copy and paste relevant sections from the manuscript (include quotes in quotation marks "like this" to indicate direct quotes from your manuscript), or elaborate on this item by providing additional information not in the ms, or briefly explain why the item is not applicable/relevant for your study

"Following the published protocol [30], this randomized controlled trial (RCT) examined the effectiveness of digital peer support intervention for psychological well-being of emerging adults by establishing evidence for implementation and clinical outcomes (refer to [30] for details of the trial). In brief, this RCT validated the applicability of harnessing the components of mattering, self-hood, compassion and mindfulness in digital peer support intervention to promote emerging adults’ mental well-being. By evaluating the mechanism of change involving digital peer support and emerging adults’ psychological functioning, this RCT provided insights into whether or how digital peer support intervened in the development of psychological symptoms. These findings may provide actionable knowledge [illegible]

Your response is too large. Try shortening some answers.

2a-ii) Scientific background, rationale: What is known about the (type of) system

Scientific background, rationale: What is known about the (type of) system that is the object of the study (be sure to discuss the use of similar systems for other conditions/diagnoses, if appropiate), motivation for the study, i.e. what are the reasons for and what is the context for this specific study, from which stakeholder viewpoint is the study performed, potential impact of findings [2]. Briefly justify the choice of the comparator.

subitem not at all important

1 ○

2 ○

3 ○

4 ○

5 ◉

essential

Clear selection

Does your paper address subitem 2a-ii? *

Copy and paste relevant sections from the manuscript (include quotes in quotation marks "like this" to indicate direct quotes from your manuscript), or elaborate on this item by providing additional information not in the ms, or briefly explain why the item is not applicable/relevant for your study

"Our review of existing literature reveals a limited understanding of how peer emotional disclosure on digital platform functions as an intervention in support of emerging adults' psychological well-being [25-27]. The administration of peer support in the offline context, specifically over face-to-face interactions, in intervening in various psychological and health outcomes among college students are well-documented [29]. Traditional (offline) peer support interventions have demonstrated consistent evidence for implementation and feasibility outcomes, user acceptability, and clinical effectiveness in mediating psychological well-being of emerging adults, particularly college students [29]. However, with regards to assessing peer support intervention for the mental health of young people, it is important to consider the mechanism of change to elucidate whether and how the [illegible]

Your response is too large. Try shortening some answers.

2b) In INTRODUCTION: Specific objectives or hypotheses

Does your paper address CONSORT subitem 2b? *

Copy and paste relevant sections from the manuscript (include quotes in quotation marks "like this" to indicate direct quotes from your manuscript), or elaborate on this item by providing additional information not in the ms, or briefly explain why the item is not applicable/relevant for your study

"This study had three primary aims. First, we evaluated the implementation effectiveness of (a) digital peer support training for emerging adults providing support (befrienders), (b) the intervention in offering an ongoing mechanism of support and (c) in identifying individuals with high risk of having a mental health condition. Second, we assessed the clinical outcomes of digital peer support training in terms of whether it (a) enhanced the four components of psychological well-being—specifically, mattering, self-hood, compassion and mindfulness and (b) improved the psychological well-being of emerging adults. Third, we investigated the mechanism of change linking digital peer support intervention to emerging adult psychological well-being."

METHODS

3a) Description of trial design (such as parallel, factorial) including allocation ratio

Does your paper address CONSORT subitem 3a? *

Copy and paste relevant sections from the manuscript (include quotes in quotation marks "like this" to indicate direct quotes from your manuscript), or elaborate on this item by providing additional information not in the ms, or briefly explain why the item is not applicable/relevant for your study

"Following the published protocol [30], this randomized controlled trial (RCT) examined the effectiveness of digital peer support intervention for psychological well-being of emerging adults by establishing evidence for implementation and clinical outcomes (refer to [30] for details of the trial)."

Your response is too large. Try shortening some answers.

3b) Important changes to methods after trial commencement (such as eligibility criteria), with reasons

Does your paper address CONSORT subitem 3b? *

Copy and paste relevant sections from the manuscript (include quotes in quotation marks "like this" to indicate direct quotes from your manuscript), or elaborate on this item by providing additional information not in the ms, or briefly explain why the item is not applicable/relevant for your study

No changes to method after trial commencement

3b-i) Bug fixes, Downtimes, Content Changes

Bug fixes, Downtimes, Content Changes: ehealth systems are often dynamic systems. A description of changes to methods therefore also includes important changes made on the intervention or comparator during the trial (e.g., major bug fixes or changes in the functionality or content) (5-iii) and other “unexpected events” that may have influenced study design such as staff changes, system failures/downtimes, etc. [2].

subitem not at all important

- 1 (selected)
- 2
- 3
- 4
- 5

essential

Clear selection

Your response is too large. Try shortening some answers.

Does your paper address subitem 3b-i?

Copy and paste relevant sections from the manuscript (include quotes in quotation marks "like this" to indicate direct quotes from your manuscript), or elaborate on this item by providing additional information not in the ms, or briefly explain why the item is not applicable/relevant for your study

No Bug fixes, Downtimes, Content Changes

4a) Eligibility criteria for participants

Does your paper address CONSORT subitem 4a? *

Copy and paste relevant sections from the manuscript (include quotes in quotation marks "like this" to indicate direct quotes from your manuscript), or elaborate on this item by providing additional information not in the ms, or briefly explain why the item is not applicable/relevant for your study

Information about eligibility criteria are in the published protocol (Yeo G, Chang W, Lee LN, Oon M, Ho D. A Digital Peer Support Platform to Translate Web-Based Peer Support for Emerging Adult Mental Well-being: Protocol for a Randomized Controlled Trial. JMIR research protocols 2022 Sep 20;11(9):e34602. [doi: 10.2196/34602)

Your response is too large. Try shortening some answers.

4a-i) Computer / Internet literacy

Computer / Internet literacy is often an implicit “de facto” eligibility criterion - this should be explicitly clarified.

subitem not at all important

1 ○

2 ○

3 ○

4 ◉

5 ○

essential

Clear selection

Does your paper address subitem 4a-i?

Copy and paste relevant sections from the manuscript (include quotes in quotation marks "like this" to indicate direct quotes from your manuscript), or elaborate on this item by providing additional information not in the ms, or briefly explain why the item is not applicable/relevant for your study

"The Acceset platform incorporates digital features, such as emotion stamps, motivation Graphic Interface Format (GIF) emotionality, and functional adjustment stickers as markers of psychological well-being—specifically, emotionality (i.e., positivity and negativity), motivations, and functional adjustment (i.e., internalizing and externalizing behaviors), respectively. During the course of the digital peer support intervention, users' engagement with these features on the platform served as a source of self-report information on their psychological well-being status. The Acceset text-based peer disclosure process begins when seekers engage the platform to seek support with managing their emotional experiences. Details on the Acceset digital peer support training, the platform and the seeker-befriender interaction (peer support workflow) detailed in the protocol that outlined the RCT [30]."

Your response is too large. Try shortening some answers.

4a-ii) Open vs. closed, web-based vs. face-to-face assessments:

Open vs. closed, web-based vs. face-to-face assessments: Mention how participants were recruited (online vs. offline), e.g., from an open access website or from a clinic, and clarify if this was a purely web-based trial, or there were face-to-face components (as part of the intervention or for assessment), i.e., to what degree got the study team to know the participant. In online-only trials, clarify if participants were quasi-anonymous and whether having multiple identities was possible or whether technical or logistical measures (e.g., cookies, email confirmation, phone calls) were used to detect/prevent these.

subitem not at all important

1 ○

2 ○

3 ○

4 ◉

5 ○

essential

Clear selection

Does your paper address subitem 4a-ii? *

Copy and paste relevant sections from the manuscript (include quotes in quotation marks "like this" to indicate direct quotes from your manuscript), or elaborate on this item by providing additional information not in the ms, or briefly explain why the item is not applicable/relevant for your study

Information is in the published protocol (Yeo G, Chang W, Lee LN, Oon M, Ho D. A Digital Peer Support Platform to Translate Web-Based Peer Support for Emerging Adult Mental Well-being: Protocol for a Randomized Controlled Trial. JMIR research protocols 2022 Sep 20;11(9):e34602. [doi: 10.2196/34602)

Your response is too large. Try shortening some answers.

4a-iii) Information giving during recruitment

Information given during recruitment. Specify how participants were briefed for recruitment and in the informed consent procedures (e.g., publish the informed consent documentation as appendix, see also item X26), as this information may have an effect on user self-selection, user expectation and may also bias results.

subitem not at all important

1 ○

2 ○

3 ○

4 ◉

5 ○

essential

Clear selection

Does your paper address subitem 4a-iii?

Copy and paste relevant sections from the manuscript (include quotes in quotation marks "like this" to indicate direct quotes from your manuscript), or elaborate on this item by providing additional information not in the ms, or briefly explain why the item is not applicable/relevant for your study

Information is in the published protocol (Yeo G, Chang W, Lee LN, Oon M, Ho D. A Digital Peer Support Platform to Translate Web-Based Peer Support for Emerging Adult Mental Well-being: Protocol for a Randomized Controlled Trial. JMIR research protocols 2022 Sep 20;11(9):e34602. [doi: 10.2196/34602)

4b) Settings and locations where the data were collected

Your response is too large. Try shortening some answers.

Does your paper address CONSORT subitem 4b? *

Copy and paste relevant sections from the manuscript (include quotes in quotation marks "like this" to indicate direct quotes from your manuscript), or elaborate on this item by providing additional information not in the ms, or briefly explain why the item is not applicable/relevant for your study

Information is in the published protocol (Yeo G, Chang W, Lee LN, Oon M, Ho D. A Digital Peer Support Platform to Translate Web-Based Peer Support for Emerging Adult Mental Well-being: Protocol for a Randomized Controlled Trial. JMIR research protocols 2022 Sep 20;11(9):e34602. [doi: 10.2196/34602)

4b-i) Report if outcomes were (self-)assessed through online questionnaires

Clearly report if outcomes were (self-)assessed through online questionnaires (as common in web-based trials) or otherwise.

subitem not at all important

1 ○

2 ○

3 ○

4 ◉

5 ○

essential

Clear selection

Your response is too large. Try shortening some answers.

Does your paper address subitem 4b-i? *

Copy and paste relevant sections from the manuscript (include quotes in quotation marks "like this" to indicate direct quotes from your manuscript), or elaborate on this item by providing additional information not in the ms, or briefly explain why the item is not applicable/relevant for your study

Information is in the published protocol (Yeo G, Chang W, Lee LN, Oon M, Ho D. A Digital Peer Support Platform to Translate Web-Based Peer Support for Emerging Adult Mental Well-being: Protocol for a Randomized Controlled Trial. JMIR research protocols 2022 Sep 20;11(9):e34602. [doi: 10.2196/34602)

4b-ii) Report how institutional affiliations are displayed

Report how institutional affiliations are displayed to potential participants [on ehealth media], as affiliations with prestigious hospitals or universities may affect volunteer rates, use, and reactions with regards to an intervention.(Not a required item – describe only if this may bias results)

subitem not at all important

- 1 (selected)
- 2
- 3
- 4
- 5

essential

Clear selection

Your response is too large. Try shortening some answers.

Does your paper address subitem 4b-ii?

Copy and paste relevant sections from the manuscript (include quotes in quotation marks "like this" to indicate direct quotes from your manuscript), or elaborate on this item by providing additional information not in the ms, or briefly explain why the item is not applicable/relevant for your study

Not a required item because this did not bias results

5) The interventions for each group with sufficient details to allow replication, including how and when they were actually administered

5-i) Mention names, credential, affiliations of the developers, sponsors, and owners

Mention names, credential, affiliations of the developers, sponsors, and owners [6] (if authors/evaluators are owners or developer of the software, this needs to be declared in a “Conflict of interest” section or mentioned elsewhere in the manuscript).

subitem not at all important

1 ○

2 ○

3 ○

4 ○

5 ◉

essential

Clear selection

Your response is too large. Try shortening some answers.

Does your paper address subitem 5-i?

Copy and paste relevant sections from the manuscript (include quotes in quotation marks "like this" to indicate direct quotes from your manuscript), or elaborate on this item by providing additional information not in the ms, or briefly explain why the item is not applicable/relevant for your study

"MO is co-founder and chief executive officer of Acceset Pte. Ltd. DH is a scientific co-founder and shareholder of KYAN Therapeutics, which is developing digital medicine-based platforms to optimize cancer therapy. DH is also an inventor of pending patents pertaining to personalized medicine."

5-ii) Describe the history/development process

Describe the history/development process of the application and previous formative evaluations (e.g., focus groups, usability testing), as these will have an impact on adoption/use rates and help with interpreting results.

subitem not at all important

1 ○

2 ○

3 ○

4 ●

5 ○

essential

Clear selection

Your response is too large. Try shortening some answers.

Does your paper address subitem 5-ii?

Copy and paste relevant sections from the manuscript (include quotes in quotation marks "like this" to indicate direct quotes from your manuscript), or elaborate on this item by providing additional information not in the ms, or briefly explain why the item is not applicable/relevant for your study

Information is in the published protocol (Yeo G, Chang W, Lee LN, Oon M, Ho D. A Digital Peer Support Platform to Translate Web-Based Peer Support for Emerging Adult Mental Well-being: Protocol for a Randomized Controlled Trial. JMIR research protocols 2022 Sep 20;11(9):e34602. [doi: 10.2196/34602)

5-iii) Revisions and updating

Revisions and updating. Clearly mention the date and/or version number of the application/intervention (and comparator, if applicable) evaluated, or describe whether the intervention underwent major changes during the evaluation process, or whether the development and/or content was “frozen” during the trial. Describe dynamic components such as news feeds or changing content which may have an impact on the replicability of the intervention (for unexpected events see item 3b).

subitem not at all important

1 (selected)

2

3

4

5

essential

Clear selection

Your response is too large. Try shortening some answers.

Does your paper address subitem 5-iii?

Copy and paste relevant sections from the manuscript (include quotes in quotation marks "like this" to indicate direct quotes from your manuscript), or elaborate on this item by providing additional information not in the ms, or briefly explain why the item is not applicable/relevant for your study

Not applicable-- the intervention did not undergo major changes during the evaluation process, neither did the development and/or content was “frozen” during the trial. No changes to dynamic components such as news feeds or changing content that may have an impact on the replicability of the intervention.

5-iv) Quality assurance methods

Provide information on quality assurance methods to ensure accuracy and quality of information provided [1], if applicable.

subitem not at all important

- 1 ○
- 2 ○
- 3 ○
- 4 ◉
- 5 ○

essential

Clear selection

Your response is too large. Try shortening some answers.

Does your paper address subitem 5-iv?

Copy and paste relevant sections from the manuscript (include quotes in quotation marks "like this" to indicate direct quotes from your manuscript), or elaborate on this item by providing additional information not in the ms, or briefly explain why the item is not applicable/relevant for your study

"Details on the trial following are listed in the published protocol that outlined the trial [30]:

- Participants and Study Setting
- Eligibility Criteria
- Peer Support Workflow
- Moderation protocol
- Data Management and Planned Analyses"

(Yeo G, Chang W, Lee LN, Oon M, Ho D. A Digital Peer Support Platform to Translate Web-Based Peer Support for Emerging Adult Mental Well-being: Protocol for a Randomized Controlled Trial. JMIR research protocols 2022 Sep 20;11(9):e34602. [doi: 10.2196/34602)

Your response is too large. Try shortening some answers.

5-v) Ensure replicability by publishing the source code, and/or providing screenshots/screen-capture video, and/or providing flowcharts of the algorithms used

Ensure replicability by publishing the source code, and/or providing screenshots/screen-capture video, and/or providing flowcharts of the algorithms used. Replicability (i.e., other researchers should in principle be able to replicate the study) is a hallmark of scientific reporting.

subitem not at all important

1 ○

2 ○

3 ○

4 ◉

5 ○

essential

Clear selection

Does your paper address subitem 5-v?

Copy and paste relevant sections from the manuscript (include quotes in quotation marks "like this" to indicate direct quotes from your manuscript), or elaborate on this item by providing additional information not in the ms, or briefly explain why the item is not applicable/relevant for your study

Acceset platform is publicly available and this Information is in the published protocol (Yeo G, Chang W, Lee LN, Oon M, Ho D. A Digital Peer Support Platform to Translate Web-Based Peer Support for Emerging Adult Mental Well-being: Protocol for a Randomized Controlled Trial. JMIR research protocols 2022 Sep 20;11(9):e34602. [doi: 10.2196/34602)

Your response is too large. Try shortening some answers.

5-vi) Digital preservation

Digital preservation: Provide the URL of the application, but as the intervention is likely to change or disappear over the course of the years; also make sure the intervention is archived (Internet Archive, webcitation.org, and/or publishing the source code or screenshots/videos alongside the article). As pages behind login screens cannot be archived, consider creating demo pages which are accessible without login.

subitem not at all important

- 1 ○
- 2 ○
- 3 ○
- 4 ○
- 5 ◉

essential

Clear selection

Does your paper address subitem 5-vi?

Copy and paste relevant sections from the manuscript (include quotes in quotation marks "like this" to indicate direct quotes from your manuscript), or elaborate on this item by providing additional information not in the ms, or briefly explain why the item is not applicable/relevant for your study

Acceset platform is publicly available and this Information is in the published protocol (Yeo G, Chang W, Lee LN, Oon M, Ho D. A Digital Peer Support Platform to Translate Web-Based Peer Support for Emerging Adult Mental Well-being: Protocol for a Randomized Controlled Trial. JMIR research protocols 2022 Sep 20;11(9):e34602. [doi: 10.2196/34602)

Your response is too large. Try shortening some answers.

5-vii) Access

Access: Describe how participants accessed the application, in what setting/context, if they had to pay (or were paid) or not, whether they had to be a member of specific group. If known, describe how participants obtained “access to the platform and Internet” [1]. To ensure access for editors/reviewers/readers, consider to provide a “backdoor” login account or demo mode for reviewers/readers to explore the application (also important for archiving purposes, see vi).

subitem not at all important

1 ○

2 ○

3 ○

4 ○

5 ◉

essential

Clear selection

Does your paper address subitem 5-vii? *

Copy and paste relevant sections from the manuscript (include quotes in quotation marks "like this" to indicate direct quotes from your manuscript), or elaborate on this item by providing additional information not in the ms, or briefly explain why the item is not applicable/relevant for your study

This Information is in the published protocol (Yeo G, Chang W, Lee LN, Oon M, Ho D. A Digital Peer Support Platform to Translate Web-Based Peer Support for Emerging Adult Mental Well-being: Protocol for a Randomized Controlled Trial. JMIR research protocols 2022 Sep 20;11(9):e34602. [doi: 10.2196/34602)

Your response is too large. Try shortening some answers.

5-viii) Mode of delivery, features/functionalities/components of the intervention and comparator, and the theoretical framework

Describe mode of delivery, features/functionalities/components of the intervention and comparator, and the theoretical framework [6] used to design them (instructional strategy [1], behaviour change techniques, persuasive features, etc., see e.g., [7, 8] for terminology). This includes an in-depth description of the content (including where it is coming from and who developed it) [1]," whether [and how] it is tailored to individual circumstances and allows users to track their progress and receive feedback" [6]. This also includes a description of communication delivery channels and – if computer-mediated communication is a component – whether communication was synchronous or asynchronous [6]. It also includes information on presentation strategies [1], including page design principles, average amount of text on pages, presence of hyperlinks to other resources, etc. [1].

subitem not at all important

1 ○

2 ○

3 ○

4 ◉

5 ○

essential

Clear selection

Your response is too large. Try shortening some answers.

Does your paper address subitem 5-viii? *

Copy and paste relevant sections from the manuscript (include quotes in quotation marks "like this" to indicate direct quotes from your manuscript), or elaborate on this item by providing additional information not in the ms, or briefly explain why the item is not applicable/relevant for your study

"Details on the Acceset digital peer support training, the platform and the seeker-befriender interaction (peer support workflow) detailed in the protocol that outlined the RCT [30]." Yeo G, Chang W, Lee LN, Oon M, Ho D. A Digital Peer Support Platform to Translate Web-Based Peer Support for Emerging Adult Mental Well-being: Protocol for a Randomized Controlled Trial. JMIR research protocols 2022 Sep 20;11(9):e34602. [doi: 10.2196/34602)

5-ix) Describe use parameters

Describe use parameters (e.g., intended "doses" and optimal timing for use). Clarify what instructions or recommendations were given to the user, e.g., regarding timing, frequency, heaviness of use, if any, or was the intervention used ad libitum.

subitem not at all important

1 ○

2 ○

3 ○

4 ○

5 ◉

essential

Clear selection

Your response is too large. Try shortening some answers.

Does your paper address subitem 5-ix?

Copy and paste relevant sections from the manuscript (include quotes in quotation marks "like this" to indicate direct quotes from your manuscript), or elaborate on this item by providing additional information not in the ms, or briefly explain why the item is not applicable/relevant for your study

This Information is in the published protocol (Yeo G, Chang W, Lee LN, Oon M, Ho D. A Digital Peer Support Platform to Translate Web-Based Peer Support for Emerging Adult Mental Well-being: Protocol for a Randomized Controlled Trial. JMIR research protocols 2022 Sep 20;11(9):e34602. [doi: 10.2196/34602)

5-x) Clarify the level of human involvement

Clarify the level of human involvement (care providers or health professionals, also technical assistance) in the e-intervention or as co-intervention (detail number and expertise of professionals involved, if any, as well as "type of assistance offered, the timing and frequency of the support, how it is initiated, and the medium by which the assistance is delivered". It may be necessary to distinguish between the level of human involvement required for the trial, and the level of human involvement required for a routine application outside of a RCT setting (discuss under item 21 – generalizability).

subitem not at all important

- 1 ○
- 2 ○
- 3 ○
- 4 ●
- 5 ○

essential

Clear selection

Your response is too large. Try shortening some answers.

Does your paper address subitem 5-x?

Copy and paste relevant sections from the manuscript (include quotes in quotation marks "like this" to indicate direct quotes from your manuscript), or elaborate on this item by providing additional information not in the ms, or briefly explain why the item is not applicable/relevant for your study

"Details on the trial following are listed in the published protocol that outlined the trial [30]:
- Participants and Study Setting
- Eligibility Criteria
- Peer Support Workflow
- Moderation protocol
- Data Management and Planned Analyses"

Yeo G, Chang W, Lee LN, Oon M, Ho D. A Digital Peer Support Platform to Translate Web-Based Peer Support for Emerging Adult Mental Well-being: Protocol for a Randomized Controlled Trial. JMIR research protocols 2022 Sep 20;11(9):e34602. [doi: 10.2196/34602

5-xi) Report any prompts/reminders used

Report any prompts/reminders used: Clarify if there were prompts (letters, emails, phone calls, SMS) to use the application, what triggered them, frequency etc. It may be necessary to distinguish between the level of prompts/reminders required for the trial, and the level of prompts/reminders for a routine application outside of a RCT setting (discuss under item 21 – generalizability).

subitem not at all important

1 ○

2 ○

3 ○

4 ●

5 ○

essential

Clear selection

Your response is too large. Try shortening some answers.

Does your paper address subitem 5-xi? *

Copy and paste relevant sections from the manuscript (include quotes in quotation marks "like this" to indicate direct quotes from your manuscript), or elaborate on this item by providing additional information not in the ms, or briefly explain why the item is not applicable/relevant for your study

"Details on the trial following are listed in the published protocol that outlined the trial [30]:
- Participants and Study Setting
- Eligibility Criteria
- Peer Support Workflow
- Moderation protocol
- Data Management and Planned Analyses"

Yeo G, Chang W, Lee LN, Oon M, Ho D. A Digital Peer Support Platform to Translate Web-Based Peer Support for Emerging Adult Mental Well-being: Protocol for a Randomized Controlled Trial. JMIR research protocols 2022 Sep 20;11(9):e34602. [doi: 10.2196/34602

5-xii) Describe any co-interventions (incl. training/support)

Describe any co-interventions (incl. training/support): Clearly state any interventions that are provided in addition to the targeted eHealth intervention, as ehealth intervention may not be designed as stand-alone intervention. This includes training sessions and support [1]. It may be necessary to distinguish between the level of training required for the trial, and the level of training for a routine application outside of a RCT setting (discuss under item 21 – generalizability.

subitem not at all important

1 ○

2 ○

3 ○

4 ●

5 ○

essential

Your response is too large. Try shortening some answers.

Does your paper address subitem 5-xii? *

Copy and paste relevant sections from the manuscript (include quotes in quotation marks "like this" to indicate direct quotes from your manuscript), or elaborate on this item by providing additional information not in the ms, or briefly explain why the item is not applicable/relevant for your study

"Details on the trial following are listed in the published protocol that outlined the trial [30]:
- Participants and Study Setting
- Eligibility Criteria
- Peer Support Workflow
- Moderation protocol
- Data Management and Planned Analyses"

Yeo G, Chang W, Lee LN, Oon M, Ho D. A Digital Peer Support Platform to Translate Web-Based Peer Support for Emerging Adult Mental Well-being: Protocol for a Randomized Controlled Trial. JMIR research protocols 2022 Sep 20;11(9):e34602. [doi: 10.2196/34602

6a) Completely defined pre-specified primary and secondary outcome measures, including how and when they were assessed

Does your paper address CONSORT subitem 6a? *

Copy and paste relevant sections from the manuscript (include quotes in quotation marks "like this" to indicate direct quotes from your manuscript), or elaborate on this item by providing additional information not in the ms, or briefly explain why the item is not applicable/relevant for your study

"Four Components of Psychological Well-Being (Mattering, Selfhood, Compassion and Mindfulness). For each letter exchange between befrienders and seekers, two undergraduate assistants extracted and coded the letter content on four components....All codes presented below were coded independently. Kappa coefficients ranged from .70 to .85, which indicated good inter-rater reliability."

"Psychological Well-Being (Times 1-4). We created a latent construct with two indicators—anxiety and depression. Befrienders and seekers provided self-report responses to the 7-item General Anxiety Disorder Questionnaire (GAD-7) ....and the PHQ-9...All the items on the GAD-7 and PHQ-9 were rated on a 4-point scale ranging from 0 (not sure at all) to 4 (nearly every day)."

Your response is too large. Try shortening some answers.

6a-i) Online questionnaires: describe if they were validated for online use and apply CHERRIES items to describe how the questionnaires were designed/deployed

If outcomes were obtained through online questionnaires, describe if they were validated for online use and apply CHERRIES items to describe how the questionnaires were designed/deployed [9].

subitem not at all important

1 ○

2 ○

3 ◉

4 ○

5 ○

essential

Clear selection

Does your paper address subitem 6a-i?

Copy and paste relevant sections from manuscript text

"Details on the trial following are listed in the published protocol that outlined the trial [30]:

- Participants and Study Setting
- Eligibility Criteria
- Peer Support Workflow
- Moderation protocol
- Data Management and Planned Analyses"

Yeo G, Chang W, Lee LN, Oon M, Ho D. A Digital Peer Support Platform to Translate Web-Based Peer Support for Emerging Adult Mental Well-being: Protocol for a Randomized Controlled Trial. JMIR research protocols 2022 Sep 20;11(9):e34602. [doi: 10.2196/34602

Your response is too large. Try shortening some answers.

6a-ii) Describe whether and how “use” (including intensity of use/dosage) was defined/measured/monitored

Describe whether and how “use” (including intensity of use/dosage) was defined/measured/monitored (logins, logfile analysis, etc.). Use/adoption metrics are important process outcomes that should be reported in any ehealth trial.

subitem not at all important

1 ○

2 ○

3 ○

4 ◉

5 ○

essential

Clear selection

Does your paper address subitem 6a-ii?

Copy and paste relevant sections from manuscript text

"Details on the trial following are listed in the published protocol that outlined the trial [30]:

- Participants and Study Setting
- Eligibility Criteria
- Peer Support Workflow
- Moderation protocol
- Data Management and Planned Analyses"

Yeo G, Chang W, Lee LN, Oon M, Ho D. A Digital Peer Support Platform to Translate Web-Based Peer Support for Emerging Adult Mental Well-being: Protocol for a Randomized Controlled Trial. JMIR research protocols 2022 Sep 20;11(9):e34602. [doi: 10.2196/34602

Your response is too large. Try shortening some answers.

6a-iii) Describe whether, how, and when qualitative feedback from participants was obtained

Describe whether, how, and when qualitative feedback from participants was obtained (e.g., through emails, feedback forms, interviews, focus groups).

subitem not at all important

1 ○

2 ○

3 ○

4 ◉

5 ○

essential

Clear selection

Does your paper address subitem 6a-iii?

Copy and paste relevant sections from manuscript text

Focused-group discussions were conducted with moderators and befrienders on the use of Acceset platform.

6b) Any changes to trial outcomes after the trial commenced, with reasons

Your response is too large. Try shortening some answers.

Does your paper address CONSORT subitem 6b? *

Copy and paste relevant sections from the manuscript (include quotes in quotation marks "like this" to indicate direct quotes from your manuscript), or elaborate on this item by providing additional information not in the ms, or briefly explain why the item is not applicable/relevant for your study

This item is not applicable because there were no changes to trial outcomes after the trial commenced

7a) How sample size was determined

NPT: When applicable, details of whether and how the clustering by care provides or centers was addressed

7a-i) Describe whether and how expected attrition was taken into account when calculating the sample size

Describe whether and how expected attrition was taken into account when calculating the sample size.

subitem not at all important

1 ○

2 ○

3 ○

4 ○

5 ◉

essential

Clear selection

Your response is too large. Try shortening some answers.

Does your paper address subitem 7a-i?

Copy and paste relevant sections from manuscript title (include quotes in quotation marks "like this" to indicate direct quotes from your manuscript), or elaborate on this item by providing additional information not in the ms, or briefly explain why the item is not applicable/relevant for your study

"Details on the trial following are listed in the published protocol that outlined the trial [30]:
- Participants and Study Setting
- Eligibility Criteria
- Peer Support Workflow
- Moderation protocol
- Data Management and Planned Analyses"

Yeo G, Chang W, Lee LN, Oon M, Ho D. A Digital Peer Support Platform to Translate Web-Based Peer Support for Emerging Adult Mental Well-being: Protocol for a Randomized Controlled Trial. JMIR research protocols 2022 Sep 20;11(9):e34602. [doi: 10.2196/34602

7b) When applicable, explanation of any interim analyses and stopping guidelines

Does your paper address CONSORT subitem 7b? *

Copy and paste relevant sections from the manuscript (include quotes in quotation marks "like this" to indicate direct quotes from your manuscript), or elaborate on this item by providing additional information not in the ms, or briefly explain why the item is not applicable/relevant for your study

"Details on the trial following are listed in the published protocol that outlined the trial [30]:
- Participants and Study Setting
- Eligibility Criteria
- Peer Support Workflow
- Moderation protocol
- Data Management and Planned Analyses"

Yeo G, Chang W, Lee LN, Oon M, Ho D. A Digital Peer Support Platform to Translate Web-Based Peer Support for Emerging Adult Mental Well-being: Protocol for a Randomized Controlled Trial. JMIR research protocols 2022 Sep 20;11(9):e34602. [doi: 10.2196/34602

Your response is too large. Try shortening some answers.

8a) Method used to generate the random allocation sequence
NPT: When applicable, how care providers were allocated to each trial group

Does your paper address CONSORT subitem 8a? *

Copy and paste relevant sections from the manuscript (include quotes in quotation marks "like this" to indicate direct quotes from your manuscript), or elaborate on this item by providing additional information not in the ms, or briefly explain why the item is not applicable/relevant for your study

"Details on the trial following are listed in the published protocol that outlined the trial [30]:
- Participants and Study Setting
- Eligibility Criteria
- Peer Support Workflow
- Moderation protocol
- Data Management and Planned Analyses"

Yeo G, Chang W, Lee LN, Oon M, Ho D. A Digital Peer Support Platform to Translate Web-Based Peer Support for Emerging Adult Mental Well-being: Protocol for a Randomized Controlled Trial. JMIR research protocols 2022 Sep 20;11(9):e34602. [doi: 10.2196/34602

8b) Type of randomisation; details of any restriction (such as blocking and block size)

Your response is too large. Try shortening some answers.

Does your paper address CONSORT subitem 8b? *

Copy and paste relevant sections from the manuscript (include quotes in quotation marks "like this" to indicate direct quotes from your manuscript), or elaborate on this item by providing additional information not in the ms, or briefly explain why the item is not applicable/relevant for your study

"Details on the trial following are listed in the published protocol that outlined the trial [30]:
- Participants and Study Setting
- Eligibility Criteria
- Peer Support Workflow
- Moderation protocol
- Data Management and Planned Analyses"

Yeo G, Chang W, Lee LN, Oon M, Ho D. A Digital Peer Support Platform to Translate Web-Based Peer Support for Emerging Adult Mental Well-being: Protocol for a Randomized Controlled Trial. JMIR research protocols 2022 Sep 20;11(9):e34602. [doi: 10.2196/34602

9) Mechanism used to implement the random allocation sequence (such as sequentially numbered containers), describing any steps taken to conceal the sequence until interventions were assigned

Does your paper address CONSORT subitem 9? *

Copy and paste relevant sections from the manuscript (include quotes in quotation marks "like this" to indicate direct quotes from your manuscript), or elaborate on this item by providing additional information not in the ms, or briefly explain why the item is not applicable/relevant for your study

"Details on the trial following are listed in the published protocol that outlined the trial [30]:
- Participants and Study Setting
- Eligibility Criteria
- Peer Support Workflow
- Moderation protocol
- Data Management and Planned Analyses"

Yeo G, Chang W, Lee LN, Oon M, Ho D. A Digital Peer Support Platform to Translate Web-Based Peer Support for Emerging Adult Mental Well-being: Protocol for a Randomized [illegible]

Your response is too large. Try shortening some answers.

10) Who generated the random allocation sequence, who enrolled participants, and who assigned participants to interventions

Does your paper address CONSORT subitem 10? *

Copy and paste relevant sections from the manuscript (include quotes in quotation marks "like this" to indicate direct quotes from your manuscript), or elaborate on this item by providing additional information not in the ms, or briefly explain why the item is not applicable/relevant for your study

"Details on the trial following are listed in the published protocol that outlined the trial [30]:
- Participants and Study Setting
- Eligibility Criteria
- Peer Support Workflow
- Moderation protocol
- Data Management and Planned Analyses"

Yeo G, Chang W, Lee LN, Oon M, Ho D. A Digital Peer Support Platform to Translate Web-Based Peer Support for Emerging Adult Mental Well-being: Protocol for a Randomized Controlled Trial. JMIR research protocols 2022 Sep 20;11(9):e34602. [doi: 10.2196/34602

11a) If done, who was blinded after assignment to interventions (for example, participants, care providers, those assessing outcomes) and how
NPT: Whether or not administering co-interventions were blinded to group assignment

Your response is too large. Try shortening some answers.

11a-i) Specify who was blinded, and who wasn’t

Specify who was blinded, and who wasn’t. Usually, in web-based trials it is not possible to blind the participants [1, 3] (this should be clearly acknowledged), but it may be possible to blind outcome assessors, those doing data analysis or those administering co-interventions (if any).

subitem not at all important

1 ○

2 ○

3 ○

4 ◉

5 ○

essential

Clear selection

Does your paper address subitem 11a-i? *

Copy and paste relevant sections from the manuscript (include quotes in quotation marks "like this" to indicate direct quotes from your manuscript), or elaborate on this item by providing additional information not in the ms, or briefly explain why the item is not applicable/relevant for your study

"Details on the trial following are listed in the published protocol that outlined the trial [30]:
- Participants and Study Setting
- Eligibility Criteria
- Peer Support Workflow
- Moderation protocol
- Data Management and Planned Analyses"

Yeo G, Chang W, Lee LN, Oon M, Ho D. A Digital Peer Support Platform to Translate Web-Based Peer Support for Emerging Adult Mental Well-being: Protocol for a Randomized Controlled Trial. JMIR research protocols 2022 Sep 20;11(9):e34602. [doi: 10.2196/34602

Your response is too large. Try shortening some answers.

11a-ii) Discuss e.g., whether participants knew which intervention was the "intervention of interest" and which one was the "comparator"

Informed consent procedures (4a-ii) can create biases and certain expectations - discuss e.g., whether participants knew which intervention was the "intervention of interest" and which one was the "comparator".

subitem not at all important

1 ○

2 ○

3 ○

4 ◉

5 ○

essential

Clear selection

Does your paper address subitem 11a-ii?

Copy and paste relevant sections from the manuscript (include quotes in quotation marks "like this" to indicate direct quotes from your manuscript), or elaborate on this item by providing additional information not in the ms, or briefly explain why the item is not applicable/relevant for your study

"Details on the trial following are listed in the published protocol that outlined the trial [30]:
- Participants and Study Setting
- Eligibility Criteria
- Peer Support Workflow
- Moderation protocol
- Data Management and Planned Analyses"

Yeo G, Chang W, Lee LN, Oon M, Ho D. A Digital Peer Support Platform to Translate Web-Based Peer Support for Emerging Adult Mental Well-being: Protocol for a Randomized Controlled Trial. JMIR research protocols 2022 Sep 20;11(9):e34602. [doi: 10.2196/34602

Your response is too large. Try shortening some answers.

11b) If relevant, description of the similarity of interventions
(this item is usually not relevant for ehealth trials as it refers to similarity of a placebo or sham intervention to a active medication/intervention)

Does your paper address CONSORT subitem 11b? *

Copy and paste relevant sections from the manuscript (include quotes in quotation marks "like this" to indicate direct quotes from your manuscript), or elaborate on this item by providing additional information not in the ms, or briefly explain why the item is not applicable/relevant for your study

This item is not applicable because this e-mental health trial as it refers to the intervention that both the intervention and wait-list control group were subjected to.

12a) Statistical methods used to compare groups for primary and secondary outcomes
NPT: When applicable, details of whether and how the clustering by care providers or centers was addressed

Does your paper address CONSORT subitem 12a? *

Copy and paste relevant sections from the manuscript (include quotes in quotation marks "like this" to indicate direct quotes from your manuscript), or elaborate on this item by providing additional information not in the ms, or briefly explain why the item is not applicable/relevant for your study

"Main Analyses: Clinical Outcomes
In addressing RQ2a and H2a, we examined if the Acceset peer support training of befrienders enhanced the four components of psychological well-being among seekers. We compared the change in mattering, self-hood, compassion and mindfulness scores of seekers over the course of the study. We conducted four sets of Latent Growth Curve Modelling (LCM) to examine the trajectories of mattering, selfhood, compassion, and mindfulness by fitting individual baseline growth models."

Your response is too large. Try shortening some answers.

12a-i) Imputation techniques to deal with attrition / missing values

Imputation techniques to deal with attrition / missing values: Not all participants will use the intervention/comparator as intended and attrition is typically high in ehealth trials. Specify how participants who did not use the application or dropped out from the trial were treated in the statistical analysis (a complete case analysis is strongly discouraged, and simple imputation techniques such as LOCF may also be problematic [4]).

subitem not at all important

1 ○

2 ○

3 ○

4 ○

5 ◉

essential

Clear selection

Does your paper address subitem 12a-i? *

Copy and paste relevant sections from the manuscript (include quotes in quotation marks "like this" to indicate direct quotes from your manuscript), or elaborate on this item by providing additional information not in the ms, or briefly explain why the item is not applicable/relevant for your study

"Preliminary analyses
The non-significant result from Little's Missing Completely at Random Test showed that the missing data involving all the variables of interest in this study (refer to Measures), which ranged from 19% to 58%, were non-systematic, $\chi^2(3783) = 766.02$, $p = 1.00$. Thus, we handled missing data using full information maximum likelihood imputation [50]. Maximum likelihood (ML) estimation is a method that ascertains the parameter values of a model using mean and variance; it maximizes the chance that the values generated are closest to those observed."

Your response is too large. Try shortening some answers.

12b) Methods for additional analyses, such as subgroup analyses and adjusted analyses

Does your paper address CONSORT subitem 12b? *

Copy and paste relevant sections from the manuscript (include quotes in quotation marks "like this" to indicate direct quotes from your manuscript), or elaborate on this item by providing additional information not in the ms, or briefly explain why the item is not applicable/relevant for your study

"Details on the trial following are listed in the published protocol that outlined the trial [30]:
- Participants and Study Setting
- Eligibility Criteria
- Peer Support Workflow
- Moderation protocol
- Data Management and Planned Analyses"

Yeo G, Chang W, Lee LN, Oon M, Ho D. A Digital Peer Support Platform to Translate Web-Based Peer Support for Emerging Adult Mental Well-being: Protocol for a Randomized Controlled Trial. JMIR research protocols 2022 Sep 20;11(9):e34602. [doi: 10.2196/34602

X26) REB/IRB Approval and Ethical Considerations [recommended as subheading under "Methods"] (not a CONSORT item)

Your response is too large. Try shortening some answers.

X26-i) Comment on ethics committee approval

subitem not at all important

1 ○

2 ○

3 ○

4 ○

5 ◉

essential

Clear selection

Does your paper address subitem X26-i?

Copy and paste relevant sections from the manuscript (include quotes in quotation marks "like this" to indicate direct quotes from your manuscript), or elaborate on this item by providing additional information not in the ms, or briefly explain why the item is not applicable/relevant for your study

"Details on the trial following are listed in the published protocol that outlined the trial [30]:
- Participants and Study Setting
- Eligibility Criteria
- Peer Support Workflow
- Moderation protocol
- Data Management and Planned Analyses"

Yeo G, Chang W, Lee LN, Oon M, Ho D. A Digital Peer Support Platform to Translate Web-Based Peer Support for Emerging Adult Mental Well-being: Protocol for a Randomized Controlled Trial. JMIR research protocols 2022 Sep 20;11(9):e34602. [doi: 10.2196/34602

Your response is too large. Try shortening some answers.

x26-ii) Outline informed consent procedures

Outline informed consent procedures e.g., if consent was obtained offline or online (how? Checkbox, etc.?), and what information was provided (see 4a-ii). See [6] for some items to be included in informed consent documents.

subitem not at all important

1 ○

2 ○

3 ○

4 ○

5 ◉

essential

Clear selection

Does your paper address subitem X26-ii?

Copy and paste relevant sections from the manuscript (include quotes in quotation marks "like this" to indicate direct quotes from your manuscript), or elaborate on this item by providing additional information not in the ms, or briefly explain why the item is not applicable/relevant for your study

"Details on the trial following are listed in the published protocol that outlined the trial [30]:

- Participants and Study Setting
- Eligibility Criteria
- Peer Support Workflow
- Moderation protocol
- Data Management and Planned Analyses"

Yeo G, Chang W, Lee LN, Oon M, Ho D. A Digital Peer Support Platform to Translate Web-Based Peer Support for Emerging Adult Mental Well-being: Protocol for a Randomized Controlled Trial. JMIR research protocols 2022 Sep 20;11(9):e34602. [doi: 10.2196/34602

Your response is too large. Try shortening some answers.

X26-iii) Safety and security procedures

Safety and security procedures, incl. privacy considerations, and any steps taken to reduce the likelihood or detection of harm (e.g., education and training, availability of a hotline)

subitem not at all important

1 ○

2 ○

3 ○

4 ○

5 ◉

essential

Clear selection

Does your paper address subitem X26-iii?

Copy and paste relevant sections from the manuscript (include quotes in quotation marks "like this" to indicate direct quotes from your manuscript), or elaborate on this item by providing additional information not in the ms, or briefly explain why the item is not applicable/relevant for your study

"Details on the trial following are listed in the published protocol that outlined the trial [30]:
- Participants and Study Setting
- Eligibility Criteria
- Peer Support Workflow
- Moderation protocol
- Data Management and Planned Analyses"

Yeo G, Chang W, Lee LN, Oon M, Ho D. A Digital Peer Support Platform to Translate Web-Based Peer Support for Emerging Adult Mental Well-being: Protocol for a Randomized Controlled Trial. JMIR research protocols 2022 Sep 20;11(9):e34602. [doi: 10.2196/34602

Your response is too large. Try shortening some answers.

13a) For each group, the numbers of participants who were randomly assigned, received intended treatment, and were analysed for the primary outcome

NPT: The number of care providers or centers performing the intervention in each group and the number of patients treated by each care provider in each center

Does your paper address CONSORT subitem 13a? *

Copy and paste relevant sections from the manuscript (include quotes in quotation marks "like this" to indicate direct quotes from your manuscript), or elaborate on this item by providing additional information not in the ms, or briefly explain why the item is not applicable/relevant for your study

"As for RQ2b and H2b, congruent with our hypothesis, engagement with Acceset digital peer support led to improved mental well-being of seekers in the intervention group, particularly lower psychological symptoms (post-intervention; $M = 7.15$, $SE = 0.88$) as compared to the waitlist control group (prior to intervention; $M = 11.75$, $SE = 0.89$), $t(89) = 3.44$, $p = 0.0009$."

13b) For each group, losses and exclusions after randomisation, together with reasons

Your response is too large. Try shortening some answers.

Does your paper address CONSORT subitem 13b? (NOTE: Preferably, this is shown in a CONSORT flow diagram) *

Copy and paste relevant sections from the manuscript (include quotes in quotation marks "like this" to indicate direct quotes from your manuscript), or elaborate on this item by providing additional information not in the ms, or briefly explain why the item is not applicable/relevant for your study

"At recruitment, we assessed the percentage of participants identified as being at an unacceptably high risk for depression and suicidality (i.e., meeting clinical cut-off of PHQ-9 > 9) and excluded them from the study. Our assessment found that seekers' (Mean = 5.89, SD = 3.69, range = 0 to 9) and befrienders' (Mean = 3.31, SD = 2.59, range = 0 to 8) depression score was below the cut-off at recruitment assessment (i.e., baseline prior to intervention). Thus, none of them were excluded from the study. During the course of the study, seekers' self-report at 3 weeks (conclusion of intervention) found that 9 of them had PHQ-9 > 9. 8 and 6 of them had scores above the clinical cut-off at 6 and 9 weeks (carry-over effect assessment), respectively. For befrienders, none of them met the clinical cut-off at 3, 6, and 9 weeks."

13b-i) Attrition diagram

Strongly recommended: An attrition diagram (e.g., proportion of participants still logging in or using the intervention/comparator in each group plotted over time, similar to a survival curve) or other figures or tables demonstrating usage/dose/engagement.

subitem not at all important

1 ○

2 ○

3 ○

4 ○

5 ◉

essential

Clear selection

Your response is too large. Try shortening some answers.

Does your paper address subitem 13b-i?

Copy and paste relevant sections from the manuscript or cite the figure number if applicable (include quotes in quotation marks "like this" to indicate direct quotes from your manuscript), or elaborate on this item by providing additional information not in the ms, or briefly explain why the item is not applicable/relevant for your study

"During the course of the study, seekers' self-report at 3 weeks (conclusion of intervention) found that 9 of them had PHQ-9 > 9. 8 and 6 of them had scores above the clinical cut-off at 6 and 9 weeks (carry-over effect assessment), respectively. For befrienders, none of them met the clinical cut-off at 3, 6, and 9 weeks. Seekers' self-report responses on the PHQ-9 functioned as the primary risk assessment for mental health conditions relating to depression and suicidality that was used to make referral to appropriate mental health support providers. These seekers were referred to appropriate mental health providers including counselling centers and hotlines within the NUS campus during the course of the study."

14a) Dates defining the periods of recruitment and follow-up

Does your paper address CONSORT subitem 14a? *

Copy and paste relevant sections from the manuscript (include quotes in quotation marks "like this" to indicate direct quotes from your manuscript), or elaborate on this item by providing additional information not in the ms, or briefly explain why the item is not applicable/relevant for your study

"Details on the trial following are listed in the published protocol that outlined the trial [30]:

- Participants and Study Setting
- Eligibility Criteria
- Peer Support Workflow
- Moderation protocol
- Data Management and Planned Analyses"

Yeo G, Chang W, Lee LN, Oon M, Ho D. A Digital Peer Support Platform to Translate Web-Based Peer Support for Emerging Adult Mental Well-being: Protocol for a Randomized Controlled Trial. JMIR research protocols 2022 Sep 20;11(9):e34602. [doi: 10.2196/34602

Your response is too large. Try shortening some answers.

14a-i) Indicate if critical “secular events” fell into the study period

Indicate if critical “secular events” fell into the study period, e.g., significant changes in Internet resources available or “changes in computer hardware or Internet delivery resources”

subitem not at all important

1 (selected)

2

3

4

5

essential

Clear selection

Does your paper address subitem 14a-i?

Copy and paste relevant sections from the manuscript (include quotes in quotation marks "like this" to indicate direct quotes from your manuscript), or elaborate on this item by providing additional information not in the ms, or briefly explain why the item is not applicable/relevant for your study

No critical “secular events” fell into the study period, e.g., significant changes in Internet resources available or “changes in computer hardware or Internet delivery resources”

14b) Why the trial ended or was stopped (early)

Your response is too large. Try shortening some answers.

Does your paper address CONSORT subitem 14b? *

Copy and paste relevant sections from the manuscript (include quotes in quotation marks "like this" to indicate direct quotes from your manuscript), or elaborate on this item by providing additional information not in the ms, or briefly explain why the item is not applicable/relevant for your study

"Details on the trial following are listed in the published protocol that outlined the trial [30]:
- Participants and Study Setting
- Eligibility Criteria
- Peer Support Workflow
- Moderation protocol
- Data Management and Planned Analyses"

Yeo G, Chang W, Lee LN, Oon M, Ho D. A Digital Peer Support Platform to Translate Web-Based Peer Support for Emerging Adult Mental Well-being: Protocol for a Randomized Controlled Trial. JMIR research protocols 2022 Sep 20;11(9):e34602. [doi: 10.2196/34602

15) A table showing baseline demographic and clinical characteristics for each group

NPT: When applicable, a description of care providers (case volume, qualification, expertise, etc.) and centers (volume) in each group

Does your paper address CONSORT subitem 15? *

Copy and paste relevant sections from the manuscript (include quotes in quotation marks "like this" to indicate direct quotes from your manuscript), or elaborate on this item by providing additional information not in the ms, or briefly explain why the item is not applicable/relevant for your study

"Table 1 and 2 present the descriptive statistics and correlations among variables for each letter exchange and at each time point, respectively. Among the four components of psychological well-being—mattering and selfhood displayed consistently moderate and positive associations, and similar associations were documented between compassion and mindfulness for both befrienders and seekers. For seekers, these four components and perceived social support had low to moderate negative associations with psychological symptoms at each time point (e.g., support and well-being at Time 1) and across time (support at Time1 and well-being at Time3)."

Your response is too large. Try shortening some answers.

15-i) Report demographics associated with digital divide issues

In ehealth trials it is particularly important to report demographics associated with digital divide issues, such as age, education, gender, social-economic status, computer/Internet/ehealth literacy of the participants, if known.

subitem not at all important

1 ○

2 ○

3 ○

4 ○

5 ◉

essential

Clear selection

Does your paper address subitem 15-i? *

Copy and paste relevant sections from the manuscript (include quotes in quotation marks "like this" to indicate direct quotes from your manuscript), or elaborate on this item by providing additional information not in the ms, or briefly explain why the item is not applicable/relevant for your study

"Details on the trial following are listed in the published protocol that outlined the trial [30]:
- Participants and Study Setting
- Eligibility Criteria
- Peer Support Workflow
- Moderation protocol
- Data Management and Planned Analyses"

Yeo G, Chang W, Lee LN, Oon M, Ho D. A Digital Peer Support Platform to Translate Web-Based Peer Support for Emerging Adult Mental Well-being: Protocol for a Randomized Controlled Trial. JMIR research protocols 2022 Sep 20;11(9):e34602. [doi: 10.2196/34602

Your response is too large. Try shortening some answers.

16) For each group, number of participants (denominator) included in each analysis and whether the analysis was by original assigned groups

16-i) Report multiple “denominators” and provide definitions

Report multiple “denominators” and provide definitions: Report N’s (and effect sizes) “across a range of study participation [and use] thresholds” [1], e.g., N exposed, N consented, N used more than x times, N used more than y weeks, N participants “used” the intervention/comparator at specific pre-defined time points of interest (in absolute and relative numbers per group). Always clearly define “use” of the intervention.

subitem not at all important

1 ○

2 ○

3 ○

4 ◉

5 ○

essential

Clear selection

Your response is too large. Try shortening some answers.

Does your paper address subitem 16-i? *

Copy and paste relevant sections from the manuscript (include quotes in quotation marks "like this" to indicate direct quotes from your manuscript), or elaborate on this item by providing additional information not in the ms, or briefly explain why the item is not applicable/relevant for your study

"On average, the waiting period for seekers to receive a response was 10.27 hours (SD = 7.61), which was within 48 hours—the acceptable response period. Specifically, the seeker drop-out rate (from both the intervention and waitlist control arms) was 4% (4 out of 100 seekers) throughout the 3-week engagement with the Acceset intervention. In total, seekers, befrienders and moderators made a total of 653 visits on the study registration website and log-on events to the platform. In total, 104 seekers, 37 befrienders, and 2 moderators registered with Acceset for the study. A total of 192 letters were exchanged between seekers and befrienders (1 exchange comprised a letter that a seeker sent and a reply from a befriender) during the 3-week intervention for both the intervention and waitlist control arms. On average, 4.67 letters were exchanged per day for 21 days (3 weeks)."

16-ii) Primary analysis should be intent-to-treat

Primary analysis should be intent-to-treat, secondary analyses could include comparing only "users", with the appropriate caveats that this is no longer a randomized sample (see 18-i).

subitem not at all important

1 ○

2 ○

3 ○

4 ●

5 ○

essential

Clear selection

Your response is too large. Try shortening some answers.

Does your paper address subitem 16-ii?

Copy and paste relevant sections from the manuscript (include quotes in quotation marks "like this" to indicate direct quotes from your manuscript), or elaborate on this item by providing additional information not in the ms, or briefly explain why the item is not applicable/relevant for your study

"We assessed the sustained effect of the digital peer support beyond the period of the intervention by evaluating the change in mental well-being of the participants in both groups (intervention and control) after 3, 6 and 9 weeks from the baseline with self-report questionnaires. In conducting independent sample t-tests, we controlled for multiple comparisons with Bonferroni's post-hoc tests."

17a) For each primary and secondary outcome, results for each group, and the estimated effect size and its precision (such as 95% confidence interval)

Does your paper address CONSORT subitem 17a? *

Copy and paste relevant sections from the manuscript (include quotes in quotation marks "like this" to indicate direct quotes from your manuscript), or elaborate on this item by providing additional information not in the ms, or briefly explain why the item is not applicable/relevant for your study

"In evaluating each model, we examined these fit indices: the χ^2 statistic, the root mean square error of approximation (RMSEA), the standardized root mean square residual (SRMR), and the comparative fit index (CFI). Acceptable model fit is indicated by RMSEA below .08, 90% CI [.05, .10], SRMR with values of 1.0 or lower, and CFI above .90. RMSEA and SRMR values smaller than .05, 90% CI [.05, .10], and CFI larger that .95 are considered good fit [53]."

Your response is too large. Try shortening some answers.

17a-i) Presentation of process outcomes such as metrics of use and intensity of use

In addition to primary/secondary (clinical) outcomes, the presentation of process outcomes such as metrics of use and intensity of use (dose, exposure) and their operational definitions is critical. This does not only refer to metrics of attrition (13-b) (often a binary variable), but also to more continuous exposure metrics such as “average session length”. These must be accompanied by a technical description how a metric like a “session” is defined (e.g., timeout after idle time) [1] (report under item 6a).

subitem not at all important

1 ○

2 ○

3 ○

4 ◉

5 ○

essential

Clear selection

Your response is too large. Try shortening some answers.

Does your paper address subitem 17a-i?

Copy and paste relevant sections from the manuscript (include quotes in quotation marks "like this" to indicate direct quotes from your manuscript), or elaborate on this item by providing additional information not in the ms, or briefly explain why the item is not applicable/relevant for your study

"On average, the waiting period for seekers to receive a response was 10.27 hours (SD = 7.61), which was within 48 hours—the acceptable response period. Specifically, the seeker drop-out rate (from both the intervention and waitlist control arms) was 4% (4 out of 100 seekers) throughout the 3-week engagement with the Acceset intervention. In total, seekers, befrienders and moderators made a total of 653 visits on the study registration website and log-on events to the platform. In total, 104 seekers, 37 befrienders, and 2 moderators registered with Acceset for the study. A total of 192 letters were exchanged between seekers and befrienders (1 exchange comprised a letter that a seeker sent and a reply from a befriender) during the 3-week intervention for both the intervention and waitlist control arms. On average, 4.67 letters were exchanged per day for 21 days (3 weeks)."

17b) For binary outcomes, presentation of both absolute and relative effect sizes is recommended

Does your paper address CONSORT subitem 17b? *

Copy and paste relevant sections from the manuscript (include quotes in quotation marks "like this" to indicate direct quotes from your manuscript), or elaborate on this item by providing additional information not in the ms, or briefly explain why the item is not applicable/relevant for your study

Not applicable because there are no binary outcomes used in this study.

18) Results of any other analyses performed, including subgroup analyses and adjusted analyses, distinguishing pre-specified from exploratory

Your response is too large. Try shortening some answers.

Does your paper address CONSORT subitem 18? *

Copy and paste relevant sections from the manuscript (include quotes in quotation marks "like this" to indicate direct quotes from your manuscript), or elaborate on this item by providing additional information not in the ms, or briefly explain why the item is not applicable/relevant for your study

"Details on the trial following are listed in the published protocol that outlined the trial [30]:
- Participants and Study Setting
- Eligibility Criteria
- Peer Support Workflow
- Moderation protocol
- Data Management and Planned Analyses"

Yeo G, Chang W, Lee LN, Oon M, Ho D. A Digital Peer Support Platform to Translate Web-Based Peer Support for Emerging Adult Mental Well-being: Protocol for a Randomized Controlled Trial. JMIR research protocols 2022 Sep 20;11(9):e34602. [doi: 10.2196/34602

18-i) Subgroup analysis of comparing only users

A subgroup analysis of comparing only users is not uncommon in ehealth trials, but if done, it must be stressed that this is a self-selected sample and no longer an unbiased sample from a randomized trial (see 16-iii).

subitem not at all important

1 (selected)

2

3

4

5

essential

Clear selection

Your response is too large. Try shortening some answers.

Does your paper address subitem 18-i?

Copy and paste relevant sections from the manuscript (include quotes in quotation marks "like this" to indicate direct quotes from your manuscript), or elaborate on this item by providing additional information not in the ms, or briefly explain why the item is not applicable/relevant for your study

Not applicable because no subgroup analysis comparing only users (i.e., intervention group)was conducted.

""We assessed the sustained effect of the digital peer support beyond the period of the intervention by evaluating the change in mental well-being of the participants in both groups (intervention and control) after 3, 6 and 9 weeks from the baseline with self-report questionnaires. In conducting independent sample t-tests, we controlled for multiple comparisons with Bonferroni's post-hoc tests."

19) All important harms or unintended effects in each group
(for specific guidance see CONSORT for harms)

Does your paper address CONSORT subitem 19? *

Copy and paste relevant sections from the manuscript (include quotes in quotation marks "like this" to indicate direct quotes from your manuscript), or elaborate on this item by providing additional information not in the ms, or briefly explain why the item is not applicable/relevant for your study

"The study team informed the counselling center on the NUS campus and referred at-risk seekers for appropriate and timely support throughout the course of the intervention, as well as at week 6 and 9 follow up assessments on all seekers (to measure carryover effects) to ensure their psychological safety. As research on mental health intervention has noted, confidentiality can circumscribe post-referral processing and follow-up of referred participants as they might not be receptive or responsive to the referral [52]."

Your response is too large. Try shortening some answers.

19-i) Include privacy breaches, technical problems

Include privacy breaches, technical problems. This does not only include physical “harm” to participants, but also incidents such as perceived or real privacy breaches [1], technical problems, and other unexpected/unintended incidents. “Unintended effects” also includes unintended positive effects [2].

subitem not at all important

1 ○

2 ○

3 ○

4 ◉

5 ○

essential

Clear selection

Does your paper address subitem 19-i?

Copy and paste relevant sections from the manuscript (include quotes in quotation marks "like this" to indicate direct quotes from your manuscript), or elaborate on this item by providing additional information not in the ms, or briefly explain why the item is not applicable/relevant for your study

"The study team informed the counselling center on the NUS campus and referred at-risk seekers for appropriate and timely support throughout the course of the intervention, as well as at week 6 and 9 follow up assessments on all seekers (to measure carryover effects) to ensure their psychological safety. As research on mental health intervention has noted, confidentiality can circumscribe post-referral processing and follow-up of referred participants as they might not be receptive or responsive to the referral [52]."

Your response is too large. Try shortening some answers.

19-ii) Include qualitative feedback from participants or observations from staff/researchers

Include qualitative feedback from participants or observations from staff/researchers, if available, on strengths and shortcomings of the application, especially if they point to unintended/unexpected effects or uses. This includes (if available) reasons for why people did or did not use the application as intended by the developers.

subitem not at all important

1 ○

2 ○

3 ○

4 ◉

5 ○

essential

Clear selection

Does your paper address subitem 19-ii?

Copy and paste relevant sections from the manuscript (include quotes in quotation marks "like this" to indicate direct quotes from your manuscript), or elaborate on this item by providing additional information not in the ms, or briefly explain why the item is not applicable/relevant for your study

Focused-group discussions were conducted with moderators and befrienders on the use of Acceset platform.

DISCUSSION

Your response is too large. Try shortening some answers.

22) Interpretation consistent with results, balancing benefits and harms, and considering other relevant evidence

NPT: In addition, take into account the choice of the comparator, lack of or partial blinding, and unequal expertise of care providers or centers in each group

22-i) Restate study questions and summarize the answers suggested by the data, starting with primary outcomes and process outcomes (use)

Restate study questions and summarize the answers suggested by the data, starting with primary outcomes and process outcomes (use).

subitem not at all important

1 ○

2 ○

3 ○

4 ○

5 ◉

essential

Clear selection

Does your paper address subitem 22-i? *

Copy and paste relevant sections from the manuscript (include quotes in quotation marks "like this" to indicate direct quotes from your manuscript), or elaborate on this item by providing additional information not in the ms, or briefly explain why the item is not applicable/relevant for your study

"This RCT assessed the implementation and clinical effectiveness of digital peer support intervention on emerging adults' psychological well-being. WOur findings provided evidence for H2b. Specifically, seekers in the intervention group had improved psychological well-being with lower symptoms of anxiety and depression at post intervention than seekers in the waitlist control group prior to intervention."

Your response is too large. Try shortening some answers.

22-ii) Highlight unanswered new questions, suggest future research

Highlight unanswered new questions, suggest future research.

subitem not at all important

1 ○

2 ○

3 ○

4 ○

5 ◉

essential

Clear selection

Does your paper address subitem 22-ii?

Copy and paste relevant sections from the manuscript (include quotes in quotation marks "like this" to indicate direct quotes from your manuscript), or elaborate on this item by providing additional information not in the ms, or briefly explain why the item is not applicable/relevant for your study

"

20) Trial limitations, addressing sources of potential bias, imprecision, and, if relevant, multiplicity of analyses

Your response is too large. Try shortening some answers.

20-i) Typical limitations in ehealth trials

Typical limitations in ehealth trials: Participants in ehealth trials are rarely blinded. Ehealth trials often look at a multiplicity of outcomes, increasing risk for a Type I error. Discuss biases due to non-use of the intervention/usability issues, biases through informed consent procedures, unexpected events.

subitem not at all important

1 ○

2 ○

3 ○

4 ●

5 ○

essential

Clear selection

Does your paper address subitem 20-i? *

Copy and paste relevant sections from the manuscript (include quotes in quotation marks "like this" to indicate direct quotes from your manuscript), or elaborate on this item by providing additional information not in the ms, or briefly explain why the item is not applicable/relevant for your study

"A possible limitation of this RCT on digital peer support intervention for emerging adult psychological well-being is the use of self-report measures for assessing clinical outcomes, which could be subjected to under- or over-estimation of anxiety and depressive symptoms. Another limitation is the preliminary results on validating the digital markers of psychological well-being on Acceset platform."

21) Generalisability (external validity, applicability) of the trial findings

NPT: External validity of the trial findings according to the intervention, comparators, patients, and care providers or centers involved in the trial

Your response is too large. Try shortening some answers.

21-i) Generalizability to other populations

Generalizability to other populations: In particular, discuss generalizability to a general Internet population, outside of a RCT setting, and general patient population, including applicability of the study results for other organizations

subitem not at all important

1 ○

2 ○

3 ○

4 ◉

5 ○

essential

Clear selection

Does your paper address subitem 21-i?

Copy and paste relevant sections from the manuscript (include quotes in quotation marks "like this" to indicate direct quotes from your manuscript), or elaborate on this item by providing additional information not in the ms, or briefly explain why the item is not applicable/relevant for your study

"Notwithstanding these limitations, this study's development and validation of a novel digital innovation realize important contributions to the field of emerging adult mental health [25, 31]. The key strengths of the proposed intervention are the scalability and sustainability of the digital peer support intervention."

Your response is too large. Try shortening some answers.

21-ii) Discuss if there were elements in the RCT that would be different in a routine application setting

Discuss if there were elements in the RCT that would be different in a routine application setting (e.g., prompts/reminders, more human involvement, training sessions or other co-interventions) and what impact the omission of these elements could have on use, adoption, or outcomes if the intervention is applied outside of a RCT setting.

subitem not at all important

1 ◯

2 ◯

3 ◯

4 ◉

5 ◯

essential

Clear selection

Does your paper address subitem 21-ii?

Copy and paste relevant sections from the manuscript (include quotes in quotation marks "like this" to indicate direct quotes from your manuscript), or elaborate on this item by providing additional information not in the ms, or briefly explain why the item is not applicable/relevant for your study

"There are potential recommendations for future design and implementation of digital peer support for youth mental health, based on results from our RCT and those from a systematic review and meta-analysis of RCTs on the sustainable effects of mental health interventions for students from IHL [66-68]."

OTHER INFORMATION

Your response is too large. Try shortening some answers.

Does your paper address CONSORT subitem 23? *

Copy and paste relevant sections from the manuscript (include quotes in quotation marks "like this" to indicate direct quotes from your manuscript), or elaborate on this item by providing additional information not in the ms, or briefly explain why the item is not applicable/relevant for your study

"Trial Registration: ClinicalTrials.gov NCT05083676"

24) Where the full trial protocol can be accessed, if available

Does your paper address CONSORT subitem 24? *

Cite a Multimedia Appendix, other reference, or copy and paste relevant sections from the manuscript (include quotes in quotation marks "like this" to indicate direct quotes from your manuscript), or elaborate on this item by providing additional information not in the ms, or briefly explain why the item is not applicable/relevant for your study

"[30] Yeo G, Chang W, Lee LN, Oon M, Ho D. A Digital Peer Support Platform to Translate Web-Based Peer Support for Emerging Adult Mental Well-being: Protocol for a Randomized Controlled Trial. JMIR research protocols 2022 Sep 20;11(9):e34602. [doi: 10.2196/34602"

25) Sources of funding and other support (such as supply of drugs), role of funders

Does your paper address CONSORT subitem 25? *

Copy and paste relevant sections from the manuscript (include quotes in quotation marks "like this" to indicate direct quotes from your manuscript), or elaborate on this item by providing additional information not in the ms, or briefly explain why the item is not applicable/relevant for your study

Full funding details are in the acknowledgement section of the manuscript

Your response is too large. Try shortening some answers.

X27) Conflicts of Interest (not a CONSORT item)

X27-i) State the relation of the study team towards the system being evaluated

In addition to the usual declaration of interests (financial or otherwise), also state the relation of the study team towards the system being evaluated, i.e., state if the authors/evaluators are distinct from or identical with the developers/sponsors of the intervention.

subitem not at all important

1 ○

2 ○

3 ○

4 ○

5 ◉

essential

Clear selection

Does your paper address subitem X27-i?

Copy and paste relevant sections from the manuscript (include quotes in quotation marks "like this" to indicate direct quotes from your manuscript), or elaborate on this item by providing additional information not in the ms, or briefly explain why the item is not applicable/relevant for your study

Full information is found under conflict of interests

About the CONSORT EHEALTH checklist

Your response is too large. Try shortening some answers.

As a result of using this checklist, did you make changes in your manuscript? *

- ○ yes, major changes
- ◉ yes, minor changes
- ○ no

What were the most important changes you made as a result of using this checklist?

Method and Results were sections where changes were made

How much time did you spend on going through the checklist INCLUDING making changes in your manuscript *

8 hours spend on going through the checklist INCLUDING making changes in your manuscript

As a result of using this checklist, do you think your manuscript has improved? *

- ◉ yes
- ○ no
- ○ Other:

Your response is too large. Try shortening some answers.

Would you like to become involved in the CONSORT EHEALTH group?

This would involve for example becoming involved in participating in a workshop and writing an "Explanation and Elaboration" document

- [x] yes
- [ ] no
- [ ] Other:

Clear selection

Any other comments or questions on CONSORT EHEALTH

Your answer

STOP - Save this form as PDF before you click submit

To generate a record that you filled in this form, we recommend to generate a PDF of this page (on a Mac, simply select "print" and then select "print as PDF") before you submit it.

When you submit your (revised) paper to JMIR, please upload the PDF as supplementary file.

Don't worry if some text in the textboxes is cut off, as we still have the complete information in our database. Thank you!

Final step: Click submit !

Click submit so we have your answers in our database!

Submit

Clear form

Never submit passwords through Google Forms.

This content is neither created nor endorsed by Google. Report Abuse - Terms of Service - Privacy Policy

Your response is too large. Try shortening some answers.
